# Solid-State Investigation of a Novel (1:2) Venlafaxine-Catechol Cocrystal: Structural, Thermal, Spectroscopic, and Solubility Studies

**DOI:** 10.1021/acsomega.6c04465

**Published:** 2026-10-08

**Authors:** Giovanna de Paula Costa, Patrícia Osório Ferreira, Guilherme Isquibola, Juliana Guedes dos Santos, Fabio Furlan Ferreira, Flávio Junior Caires

**Affiliations:** † School of Sciences, São Paulo State University, Bauru 17033-360, SP, Brazil; ‡ LCCEM/CCNH, Federal University of ABC, Santo André 09280-560, SP, Brazil; § Institute of Chemistry, São Paulo State University, Araraquara 14800-900, SP, Brazil

## Abstract

This work reports the third cocrystal of venlafaxine (VLF), expanding the limited database of multicomponent solid forms of this clinically important antidepressant. A systematic cocrystal screening is conducted using a mechanochemical method and catechol (CAT) as coformer. The structure of the novel cocrystal is successfully solved from powder X-ray diffraction data, providing detailed insights into its crystal lattice and revealing a venlafaxine-catechol cocrystal in 1:2 stoichiometry. Hirshfeld surface analysis is employed to characterize the distribution and nature of intermolecular interactions within the cocrystal. A binary phase diagram of the system shows the formation of one cocrystal and two eutectic mixtures at different stoichiometric ratios (7:3 and 1:4 (*n*/*n*)). All solid forms are further characterized by DSC, PXRD, and FTIR spectroscopy, confirming their distinct structural and chemical features. Solubility studies show that the cocrystal exhibits the highest solubility in comparison to the eutectic mixtures and pure VLF, while the eutectics also enhance solubility relative to the VLF. These results indicate that the enhanced solubilization of the eutectic systems results from the combined effects of CAT-mediated solubilization and solid-state transformations, including in situ formation of the (1:2) VLF-CAT cocrystal, with the extent of these processes depending on the initial solid-state composition. The identification of a novel (1:2) VLF-CAT cocrystal, alongside the characterization of eutectic mixtures, highlights the relevance of cocrystallization strategies for improving the bioavailability, physical stability, and potential therapeutic performance of poorly water-soluble drugs. These findings provide a framework for the rational design of multicomponent solid forms, with implications for future pharmaceutical development and optimization of drug formulations.

## Graphical abstract

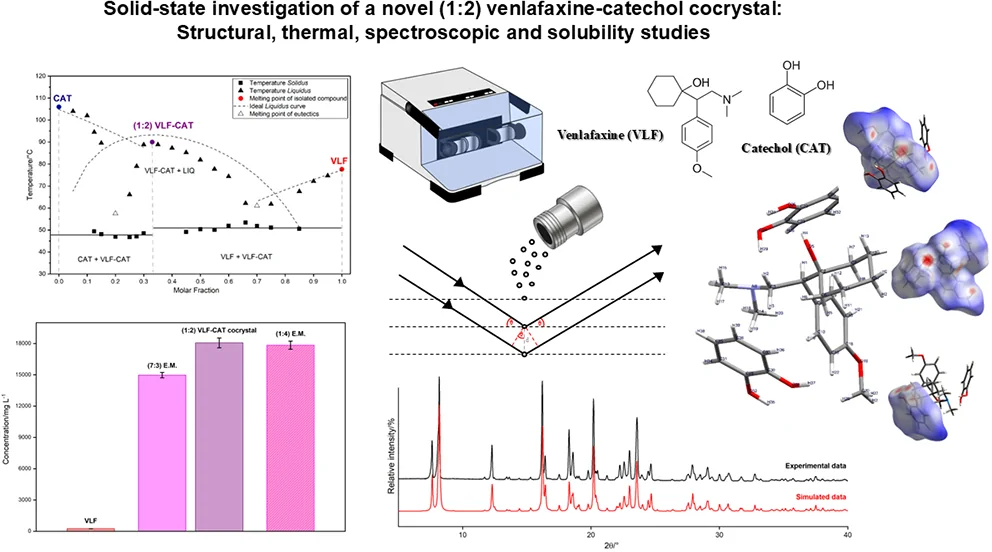

## 1 Introduction

Solid forms are an efficient strategy to improve the properties of active pharmaceutical ingredients (APIs), including single-component systems such as polymorphs and amorphous forms, as well as multicomponent systems such as salts, solvates, coamorphous systems, eutectic mixtures, and cocrystals.^1,2^

Among these, eutectic mixtures and cocrystals are closely related because they may coexist within the same binary phase diagram.^3–6^ However, they differ significantly in their structural organization and intermolecular interactions.^6^ Eutectic mixtures are multicomponent systems composed of two or more crystalline phases that are immiscible in the solid state. Therefore, each component retains its own crystal structure, while the overall system exhibits a lower melting temperature than the individual constituents.^6,7^ In contrast, cocrystals are crystalline single-phase solids composed of two or more distinct molecular or ionic compounds, typically present in a stoichiometric ratio, that are neither simple salts nor solvates.^1^ They are formed through noncovalent interactions in the solid state, such as hydrogen bonding, π-π stacking, and van der Waals forces, generally without proton transfer between the interacting species.^8–10^

Cocrystallization has attracted considerable interest in both academia and the pharmaceutical industry as an effective strategy to improve the bioavailability of poorly water-soluble drugs.^11^ In addition to enhancing solubility and dissolution, cocrystallization can improve physical and mechanical properties of pharmaceutical solids without altering the pharmacological activity of the API.^12^ Furthermore, cocrystals composed of two APIs enable combination therapy through a single dosage form, potentially improving patient adherence while allowing simultaneous drug delivery and, in some cases, reducing the doses required to achieve the desired therapeutic effect.^11,13,14^

Venlafaxine (VLF) is a well-established antidepressant that has been on the market for over 30 years and is widely prescribed for the treatment of major depressive disorder, panic disorder, and generalized anxiety disorder.^15,16^ However, VLF exhibits poor water solubility, which limits its bioavailability. Consequently, crystal engineering strategies have been employed to develop new multicomponent solid forms to improve their physicochemical properties.^17,18^

According to the Cambridge Structural Database (CSD), only a limited number of venlafaxine salts have been reported. These include the widely used hydrochloride and hydrobromide salts, as well as salts with besylate monohydrate, several carboxylic acids (caffeic, phthalic, 4-chlorobenzoic, maleic, coumaric, ferulic, oxalic, fumaric, salicylic, and 3,5-dinitrobenzoic acids), the diastereomeric salts with O,O′-di-p-toluoyl-(R,R)-tartaric acid, and the saccharin salt.^17–25^ In contrast, only two venlafaxine cocrystals have been reported to date: a neutral cocrystal with celecoxib and a (1:2) salt cocrystal with 3,5-dinitrobenzoic acid.^17,26^ This marked disparity highlights the limited structural diversity of venlafaxine cocrystals compared with its salt forms.

Catechol (CAT) has attracted considerable interest as a coformer in crystal engineering owing to its ability to establish a wide range of intermolecular interactions, particularly hydrogen bonds, which promote the formation and stabilization of multicomponent solid forms. Previous studies have demonstrated the successful use of catechol as a coformer for pharmaceutical compounds, highlighting its potential for the design of novel solid forms with tailored structural and physicochemical properties.^27–30^ However, its potential pharmaceutical application must also be evaluated in light of its toxicological profile. Catechol can undergo oxidative transformations, generating reactive semiquinone radicals and quinone species that may participate in redox cycling and induce oxidative stress.^31,32^ Therefore, although catechol is an attractive coformer from a crystal engineering perspective, its incorporation into pharmaceutical multicomponent systems requires careful assessment of its safety profile and the associated benefit–risk balance.

In this work, we report the third venlafaxine cocrystal, named (1:2) VLF-CAT, and two eutectic mixtures, named (7:3) E.M. and (1:4) E.M., addressing this gap in the literature and providing new insights into the design of multicomponent solid forms to enhance the physicochemical properties of this clinically important antidepressant. This work provides not only a new cocrystal form but also insights into the rational design of multicomponent solids for improving the performance of poorly soluble antidepressants.

## 2 Experimental Section

### 2.1 Materials

Venlafaxine hydrochloride (purity ≥ 99.0%) was purchased from a local drug store (São Carlos, São Paulo—Brazil), and catechol (purity ≥ 99.0%) was purchased from Sigma-Aldrich (St. Louis, Missouri—United States). Ethyl acetate (EtOAC, purity ≥ 99.0%) was purchased from Neon Reagentes (Suzano, São Paulo—Brazil). The venlafaxine free base (VLF) was prepared using literature procedures^22,25^ and corresponds to the polymorphic form I, as determined by PXRD (Supporting Information (SI), Figure S1).^33^ To date, there are no reports of CAT polymorphism.^27^

### 2.2 Methods

#### 2.2.1 Cocrystal and Eutectic Mixtures Synthesis

Cocrystal was initially screened using mechanochemical methods, including liquid-assisted grinding (LAG) and neat grinding (NG) at 15 and 30 Hz for 30 min. The synthesis was carried out in a Retsch MM 400 mill (Germany) using 10 mL stainless steel grinding jars containing one ball of the same material (Φ = 7 mm). The cocrystal and eutectic mixtures were prepared by milling the starting materials at 30 Hz for 30 min, using molar ratios of 1:2, 7:3, and 1:4, respectively (total mass of 150 mg). A physical mixture of the precursors was prepared by manually mixing in an agate mortar the starting materials in a 1:2 molar ratio for 5 min. The LAG approach was explored using EtOAC (η = 0.25 μL mg^–1^), as reported in the literature.^34^

The samples were stored in desiccators over anhydrous calcium chloride at room temperature, and PXRD analyses were periodically performed to verify the integrity of their crystal structures (Figure S2).

Several attempts were made to obtain single crystals suitable for single-crystal X-ray diffraction analysis, however, all were unsuccessful. Crystal growth was hindered by the significant solubility difference between venlafaxine and catechol under the investigated conditions. Consequently, crystals of sufficient size and quality for structural analysis could not be obtained.

#### 2.2.2 Powder X-ray Diffraction (PXRD) and Structure Determination

The samples were analyzed in a MiniFlex diffractometer (Rigaku, Japan). The diffractograms were obtained using a copper tube operated at 40 kV and 15 mA, with Cu*K*α radiation (λ = 1.5418 Å), 5° ≤ 2θ ≤ 40°, and a scanning rate of 5° min^–1^ in continuous-scanning mode.

The (1:2) VLF-CAT cocrystal structure was solved from PXRD data, which was acquired at room temperature on a STADI-P powder diffractometer (Stoe, Darmstadt, Germany) in transmission geometry and using monochromatic Cu*K*α_1_ radiation (λ = 1.54056 Å) obtained by a Ge (111) curved monochromator with the X-ray tube operating at 40 kV and 40 mA. The diffraction pattern was recorded using a Mythen 1 K (Dectris, Baden, Switzerland) detector over the angular range 2.000°–93.335° (2θ), with a step size of 0.015° and an integration time of 100 s at each 1.05°. Indexing and Rietveld refinement of the PXRD data were performed using the TOPAS-Academic v.7 software,^35^ and the detailed procedure is described elsewhere.^36–38^ The structure was indexed using the DASH software, which utilized 3D drawings of both VLF and CAT obtained from the Crystallographic Information Framework (CIF) files available at the Cambridge Crystallographic Data Centre (CCDC). These drawings were then used in a simulated annealing algorithm, which was also implemented within the DASH software.^39^ The best result obtained was then utilized for the Rietveld refinement. The crystal data and details of measurements for all compounds are reported in Table 1, and the crystallographic tables are presented in the Supporting Information (Tables 3–6). The crystallographic CIF file for this study has been deposited in the Cambridge Crystallographic Data Centre (CCDC) under deposition number 2530552. These data can be obtained free of charge via www.ccdc.cam.ac.uk/data_request/cif

**Table 1:** Crystallographic Data and Structure Refinement Details

Compound	(1:2) VLF-CAT Cocrystal
Empirical formula	C_29_H_39_NO_6_
Formula weight	497.61
*T* (K)	298(2)
Crystal system	Triclinic
Space group	*P*1̅
*a* (Å)	9.5442(5)
*b* (Å)	12.2687(10)
*c* (Å)	13.0228(8)
α (deg.)	117.198(7)
β (deg.)	92.346(5)
γ (deg.)	90.291(7)
Volume (Å^3^)	1354.68(18)
*Z*	2
*Z*′	1
Calculated density (g cm^–3^)	1.2200
*F*(000)	536
Radiation type	Cu*K*α_1_
Wavelength/Å	1.54056
Diffractometer	STADI-P diffractometer
2θ range (°)	2.000–93.335°
Step size (°)	0.015
Refinement method	Rietveld refinement
Number of refined parameters	218
Number of restraints	89
*R*_p_ (%)	4.19
*R*_wp_ (%)	5.57
*R*_exp_ (%)	3.67
GOF (χ^2^)	1.556
CCDC number	2530552

#### 2.2.3 Hirshfeld Surface Analysis (HS)

The intermolecular interactions were investigated using HS analysis and 2D-fingerprint plots in CrystalExplorer17.^40^

The HS were generated from the crystallographic information file (CIF) obtained by the structure solution from PXRD patterns. The surfaces were mapped using the normalized contact distance (*d*_norm_), which accounts for the internal (*d*_*i*_) and external (*d*_*e*_) distances from the surface to the nearest nucleus scaled by their respective van der Waals radii.^41,42^

#### 2.2.4 TG-DTA Analysis

Simultaneous thermogravimetry and differential thermal analysis (TG-DTA) were carried out using a NETZSCH STA 449 Jupiter Instrument (Erich NETZSCH GmbH & Co. Holding KG, Germany). Samples (approximately 5.0 mg) were placed in α-Al_2_O_3_ crucibles (70 μL) and heated from 30 to 800 °C at a rate of 10 °C min^–1^ under a dry air purge (50 mL min^–1^).

#### 2.2.5 DSC Analysis

Differential scanning calorimetry (DSC) curves were obtained using the METTLER TOLEDO DSC 1 STARe System (Zurich, Switzerland) from 25 °C to the thermal stability limit of the compounds. Samples (approximately 2.5 mg) were placed in 40 μL aluminum crucibles sealed with aluminum lids without a central hole to improve the resolution of thermal events. Analyses were performed at a heating rate of 10 °C min^–1^ under dry air as purge gas, at a flow rate of 50 mL min^–1^. The onset and peak temperatures were obtained using STARe (version 16.00b).

The binary phase diagram was constructed using DSC data (*T*_onset_ and *T*_peak_),^43^ obtained from a series of VLF-CAT mixtures with different molar fractions (0.95, 0.90, 0.85, 0.75, 0.70, 0.65, 0.60, 0.55, 0.50, 0.45, 0.40, 0.35, 0.33, 0.30, 0.275, 0.25, 0.20, 0.15, 0.125, 0.10, 0.05). These mixtures were prepared by LAG at 30 Hz for 30 min.

#### 2.2.6 Infrared Spectroscopy

FTIR spectra were recorded using a PerkinElmer Spectrum Two spectrometer (PerkinElmer, Shelton, CT, USA) equipped with a diamond attenuated total reflectance (ATR) accessory. Spectra were collected over the range of 4000–550 cm^–1^ with a spectral resolution of 4 cm^–1^ and 32 scans per spectrum.

#### 2.2.7 Solubility Studies

The solubility of VLF and the prepared multicomponent solids was determined using the saturation shake-flask method. 50 mg of VLF powder equivalents were homogenized in an agate mortar and added to test tubes containing 5 mL of acetate buffer medium (pH 4.5). The samples were placed in a Marconi shaker incubator (Piracicaba, São Paulo—Brazil, model MA 410/CF) with orbital agitation at 160 rpm for 48 h at 37 ± 0.5 °C. Each compound was performed in triplicate.

After shaker time, each sample was filtered through a 0.22 mm PTFE membrane and appropriately diluted in acetate buffer medium. The samples were quantified using an Agilent Technologies UV–Vis spectrophotometer (Santa Clara, California—United States), model Cary 8454, with quartz cuvettes (1.0 cm path length) and a maximum wavelength (λ_max_) of 225 nm.

Selectivity data and method descriptions, including analytical curves and PXRD analysis of the remaining solids, are available in Supporting Information. The selectivity of the analytical method was evaluated by comparing the UV–Vis spectra of the drug (VLF), the coformer (CAT), and the obtained compounds ((7:3) E.M., (1:2) VLF-CAT cocrystal, (1:4) E.M.) (Figure S7). The analysis revealed significant overlaps between the absorption spectra of VLF and CAT, indicating interference from the coformer signal and precluding direct quantification of Venlafaxine in the studied systems. To overcome this limitation, the quantification was performed using a mathematical deconvolution approach based on simultaneous equations derived from the Beer–Lambert law applied to the spectrophotometric signals.^44^ This mathematical deconvolution and analytical curves data are available in the Supporting Information (Tables S8 and S10 and Figure S9­(a,b)).

## 3 Results and Discussion

### 3.1 Cocrystal and Eutectic Mixtures Screening

Potential coformers were selected based on crystal engineering principles, taking into account the Δp*K*_a_ between the components, the presence of complementary hydrogen-bond donor and acceptor functional groups, and molecular geometry. More than 20 candidate coformers, including amines, phenolic compounds and other pharmaceutical compounds, were evaluated during the screening process. Among the systems investigated, catechol was identified as the most suitable coformer for the formation of a novel multicomponent solid with venlafaxine.

A mechanochemical method was employed to synthesize the multicomponent solids analyzed. Liquid-assisted grinding (LAG, using EtOAc) and neat grinding (NG) were evaluated at 15 and 30 Hz, with a fixed milling time of 30 min.

The DSC curves (Figure 1(a)) show that the use of EtOAc and higher milling frequencies favors the formation of the (1:2) VLF-CAT cocrystal. This is evidenced by the DSC curve of the VLF-CAT/LAG sample prepared at 30 Hz, which exhibits a single endothermic event at approximately 90 °C, corresponding to the melting of the cocrystal. In contrast, the sample prepared by NG at 30 Hz exhibits a slightly lower melting temperature (88 °C). This behavior is attributed to the greater mechanical stress imposed during solvent-free milling, which can introduce crystal defects and reduce crystallinity, thereby resulting in a slight decrease in the melting temperature.^45^ This interpretation is supported by the PXRD results (Figure S11), which show that the samples prepared by LAG and NG at 30 Hz display the same diffraction pattern, confirming the formation of the same crystalline phase.

**Figure 1:**
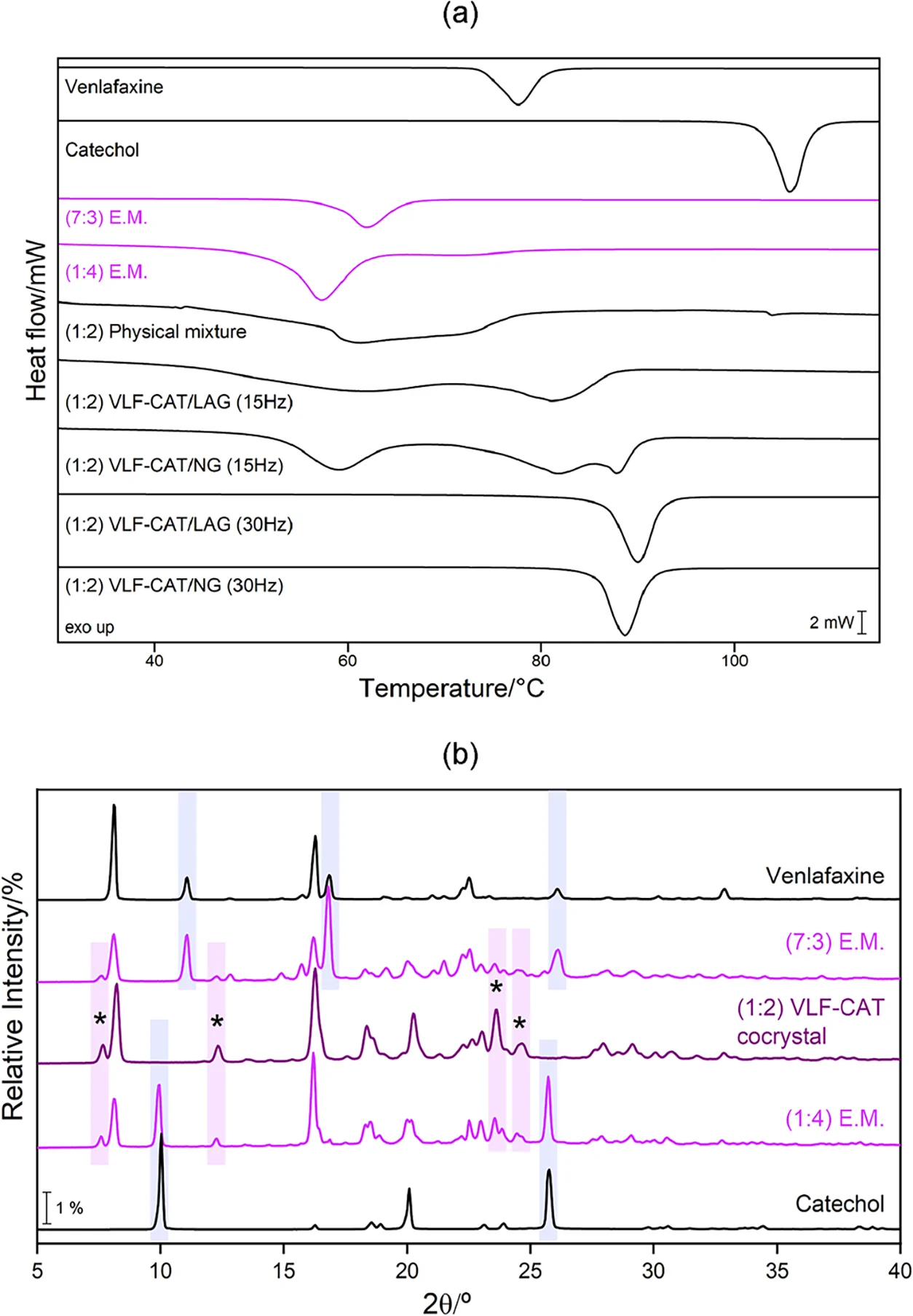
(a) DSC curves of venlafaxine, catechol, the eutectic mixtures (E.M.), (1:2) physical mixture (PM), and the products obtained by liquid-assisted grinding (LAG) and neat grinding (NG) at 15 and 30 Hz for 30 min and (b) PXRD patterns of venlafaxine, catechol, the eutectic mixtures (E.M.), and the (1:2) VLF-CAT cocrystal. Notes: Shaded regions indicate characteristic reflections of the starting materials, whereas asterisks (*) denote new diffraction peaks associated with the (1:2) VLF–CAT cocrystal.

The PXRD patterns of venlafaxine, catechol, the (1:2) VLF–CAT cocrystal, and the eutectic mixtures are shown in Figure 1(b). The cocrystal exhibits a distinct diffraction pattern compared with those of the starting materials, confirming the formation of a new crystalline phase. In contrast, the PXRD patterns of the (7:3) and (1:4) eutectic mixtures contain only the characteristic reflections of the parent compounds. Although differences in relative peak intensities are observed due to the varying phase proportions, no new diffraction peaks are detected, indicating that the crystal structures of both components remain unchanged. These findings are in excellent agreement with the DSC results, confirming that the 1:2 composition corresponds to a new cocrystal phase, whereas the eutectic compositions consist solely of the coexistence of the individual crystalline phases of venlafaxine and catechol.

Samples prepared under the mechanochemical conditions show additional endothermic events, indicating the presence of unreacted material despite the use of the appropriate stoichiometric composition. The samples prepared by LAG and NG at 15 Hz show an endothermic event around 58 °C, corresponding to the melting of the VLF-CAT eutectic composition. A second endothermic event, observed for both LAG and NG samples, is attributed to the melting of unreacted venlafaxine (81 °C), whereas a third endothermic event, detected only for the NG sample, corresponds to the melting of the cocrystal phase (87 °C). The physical mixture of the precursors exhibits evidence of solid-state interactions,

The physical mixing of the precursors shows evidence of solid-state interactions, with an extended endothermic event between 60 and 70 °C and the melting of the unreacted coformer at around 103 °C. Microscopy images and video of the contact zone between VLF and CAT over 10 min show the onset of interaction between the components (Figure S12, Video S13). Particle mobility and signs of solid-state diffusion are observed, consistent with the induction phase preceding nucleation at the contact zone (red boxes), as typically described in the Kofler contact method for studying solid interactions.^46–48^

### 3.2 Crystal Structure Analysis

The crystal structure was solved and refined in the triclinic space group *P*1̅ with *Z* = 2. The asymmetric unit (Figure 2(a)) contains one VLF molecule and two CAT molecules corresponding to the 1:2 stoichiometric ratio of the cocrystal. In the crystal structure, VLF and CAT are linked by intermolecular O–H···N and O–H···O hydrogen bonds, forming supramolecular synthons (Figure 2(b)). These interactions propagate through inversion symmetry to form a centrosymmetric three-dimensional hydrogen-bonded network (Figure 2(b)).

**Figure 2:**
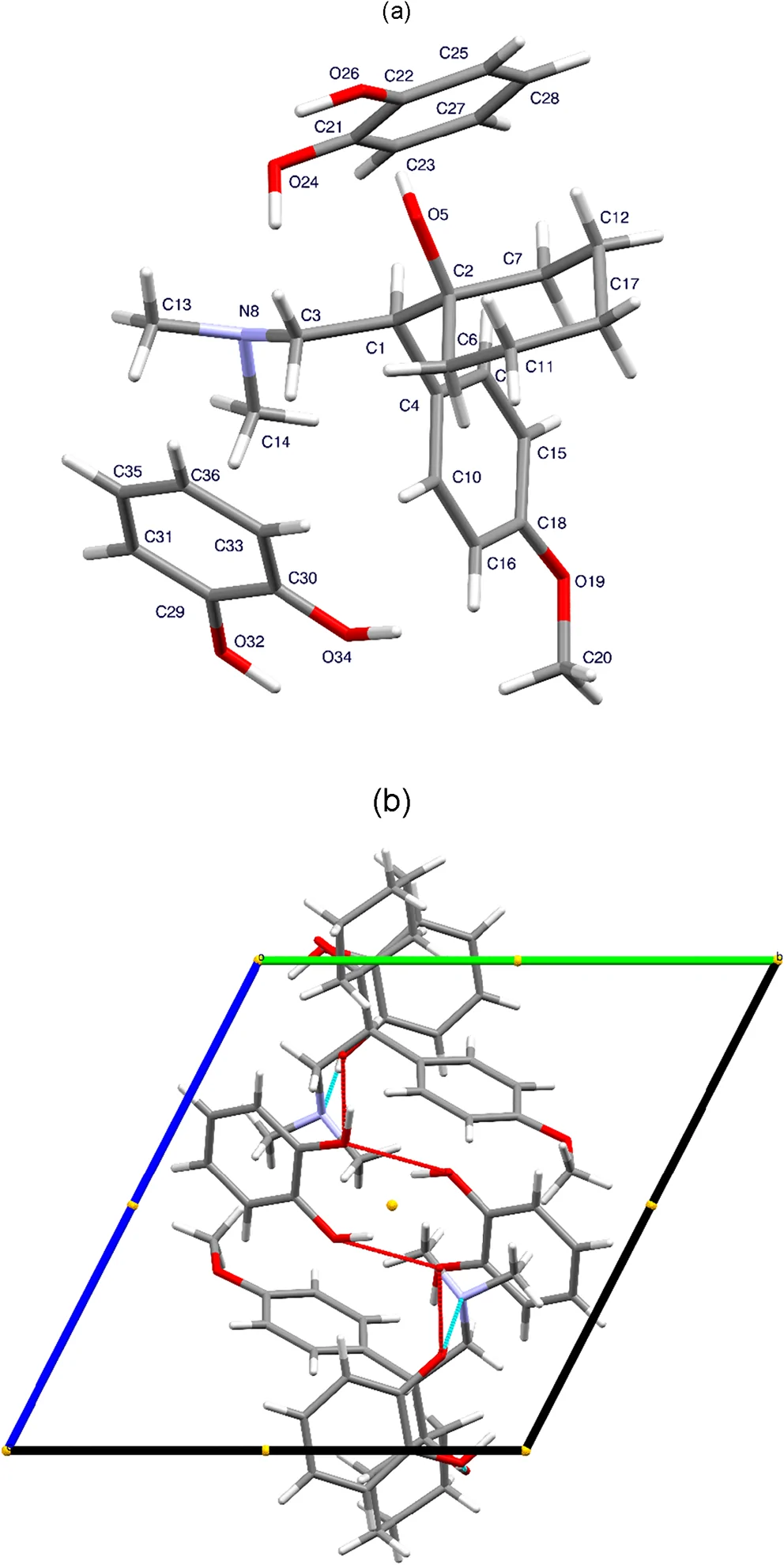
(a) Asymmetric unit of the (1:2) VLF-CAT cocrystal showing atom labeling and (b) crystal packing diagram of the (1:2) VLF-CAT cocrystal viewed along the *a* axis, showing the hydrogen-bonded supramolecular network.

The crystallographic data of the (1:2) VLF-CAT cocrystal are summarized in Table 1, and the hydrogen-bond geometries and parameters are listed in Table 2.

**Table 2:** Crystallographic Hydrogen-Bond Geometries and Parameters

D—H···A	H···A (Å)	D···A (Å)	∠D—H···A (°)	Symmetry code
O24–H29···N8	1.87(3)	2.83(3)	163(3)	–*x*, – *y*, −*z*
O5–H4···O26	1.87(3)	2.85(3)	169(2)	–*x*, −*y*, −*z*

#### 3.3 Hirshfeld Surface Analysis (HS)

The HS of all fragments was generated from crystallographic data and represents the region where the electron density of a molecule interacts with that of its neighboring molecules. This analysis is widely used to investigate intermolecular interactions and to better understand crystal packing in multicomponent solids.^17,49^ In the HS mapped over *d_*norm, contacts shorter and longer than the van der Waals radii are shown in red and blue, respectively, while contacts near the van der Waals distances are shown in white. The surface highlights the contribution of intermolecular interactions to the stability of the crystal structure. In particular, the red spots observed on the surface indicate regions of strong intermolecular interactions.^50,51^

The 2D fingerprint analysis shows that most intermolecular interactions are present across all fragments, with H···H contacts contribute the largest percentage of observed interactions (Figure 3).

**Figure 3:**
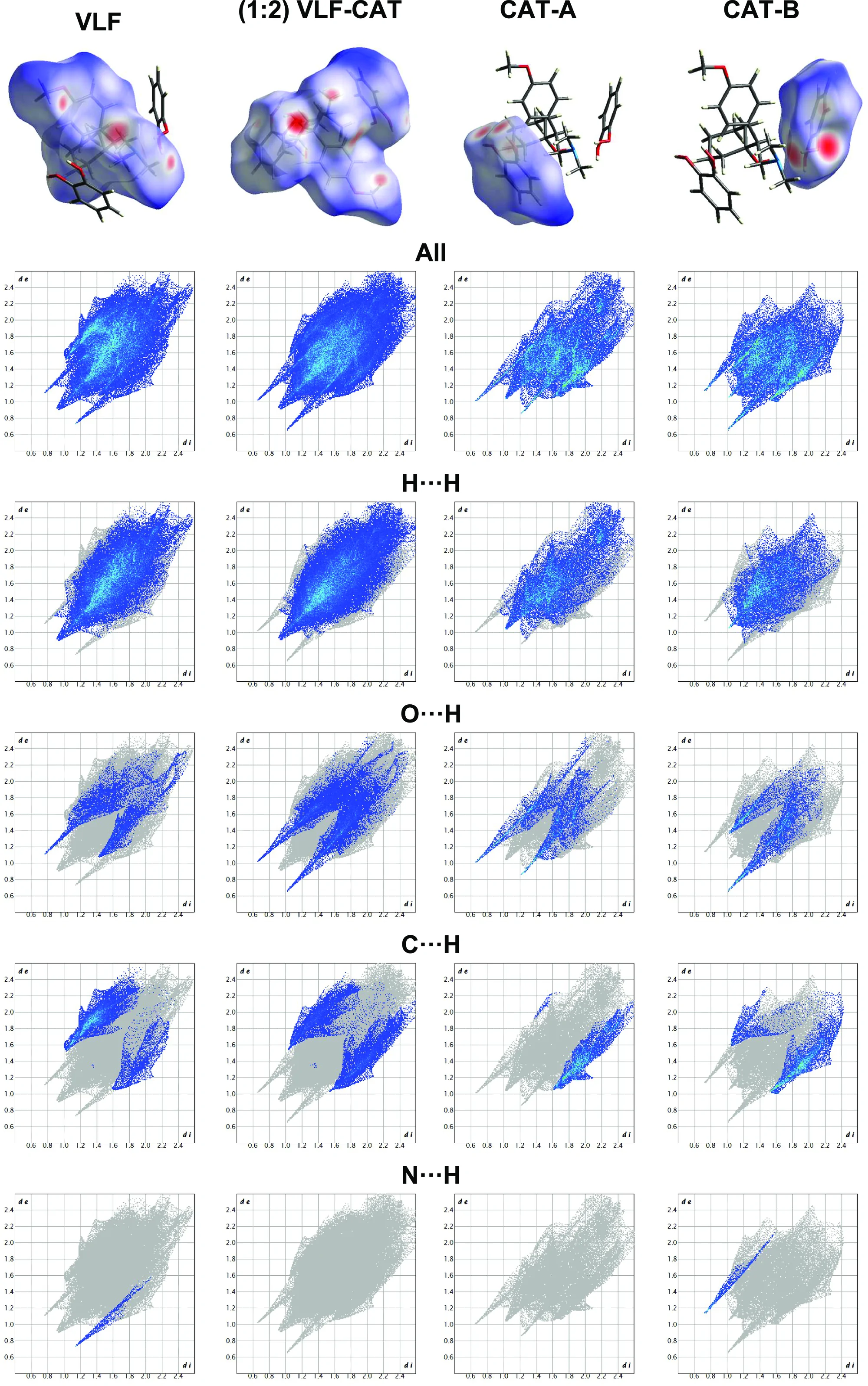
Hirshfeld surface mapped over d_norm highlighting intermolecular interactions in the VLF, (1:2) VLF-CAT, CAT-A, and CAT-B.

The fingerprint plots for O···H/H···O contacts display characteristic sharp spikes, indicating strong hydrogen-bond interactions (Figure 3).

These interactions arise mainly from O–H···O and O–H···N hydrogen bonds involving the hydroxyl groups of catechol and the venlafaxine molecule. The slightly different contributions observed for CAT-A and CAT-B reflect their distinct hydrogen-bonding environments within the crystal packing.

The C···H contacts correspond to weaker interactions (Figure 3), likely associated with aromatic contacts such as C–H···π interactions.

Finally, N···H contacts are observed for VLF and CAT-B with minor contributions but still display sharp peaks in the fingerprint plots (Figure 3), indicating hydrogen-bond interactions that may be attributed to contacts between catechol and the nitrogen atom of the venlafaxine molecule. Overall, the Hirshfeld surface analysis provides a clear visualization of the intermolecular interactions responsible for the stabilization and packing of the (1:2) VLF-CAT cocrystal. The percentage contributions of intermolecular contacts to the Hirshfeld surface are available on Figure 4.

**Figure 4:**
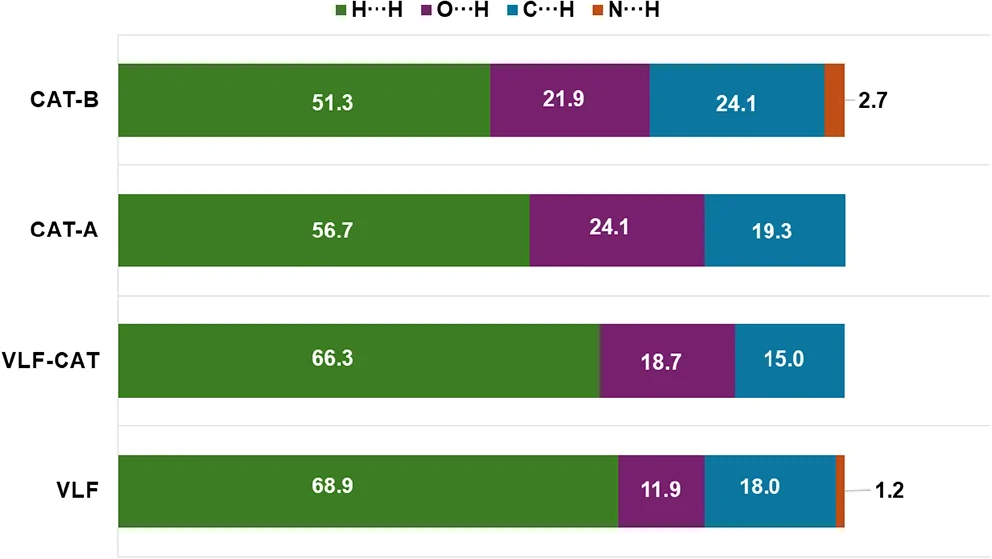
Percentage contributions of intermolecular contacts to the Hirshfeld surface.

### 3.4 Cocrystal Characterization

DSC curves (Figure 5(a)) were obtained from 30 to 115 °C under the experimental conditions described in the Methodssection.

**Figure 5:**
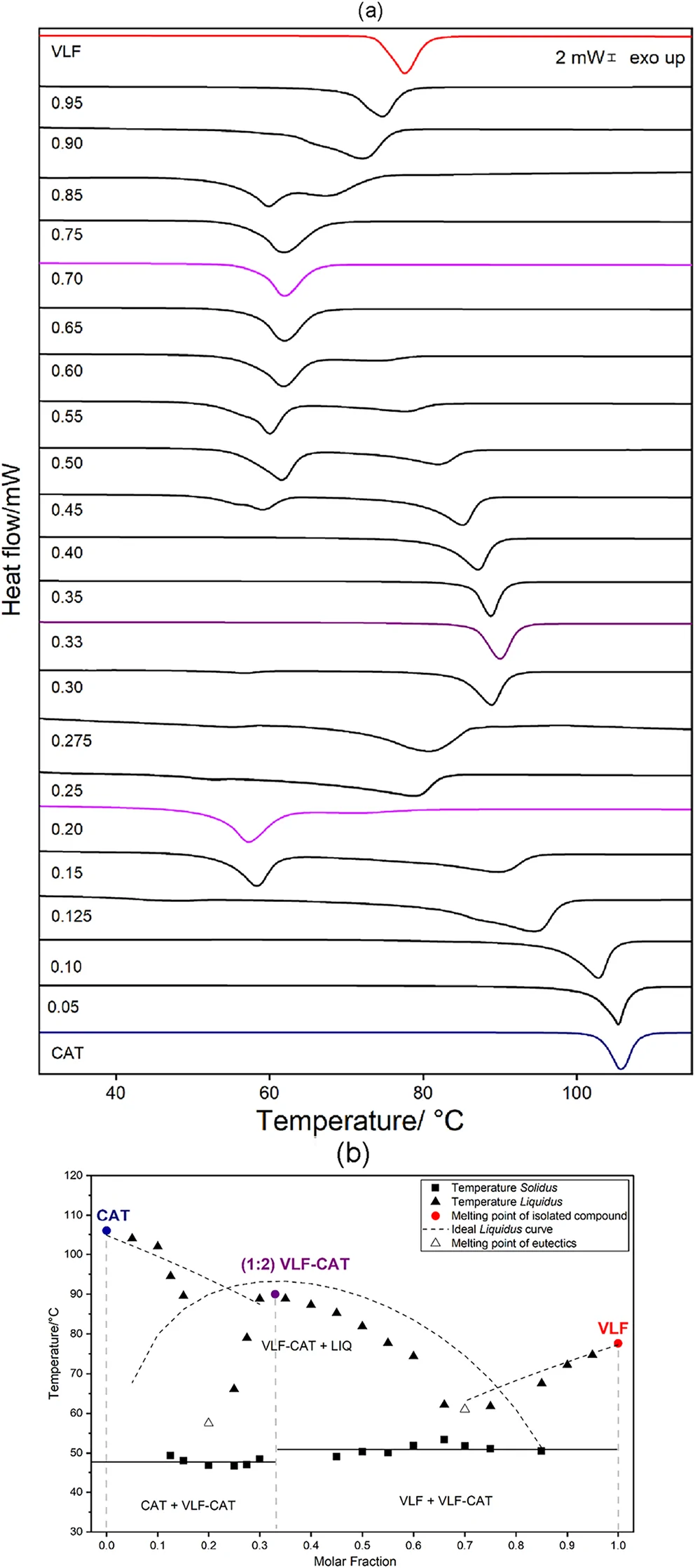
(a) DSC heating curves of VLF-CAT binary mixtures with different molar fractions, prepared by mechanochemistry (LAG, 30 Hz and 30 min); (b) Binary phase diagram of the (1:2) VLF-CAT cocrystal.

The Schröder-Van Laar’s equation was used to plot ideal liquidus curves of VLF and CAT (Figure 5(b); dotted line), being that the melting enthalpy remained constant and the solid phases were completely immiscible.^3,52^ To account for the nonequimolar stoichiometry of the (1:2) VLF-CAT cocrystal, a modified form of the Prigogine–Defay’s equation^3,53^ (eq 1) was applied to model the cocrystal ideal liquidus line (Figure 5(b); dotted line).

1 T=(1Tc−RΔfH°{ln[x2l((1−x2l)^2)]−ln⁡0.125})−1

The experimental solidus (Figure 5(b); black square) and liquidus (Figure 5(b); black triangle) curves were determined based on the *T*_onset_ of the first peaks and the *T*_peak_ of the second peaks, respectively.

The binary phase diagram, plotted as molar fraction (*x*-axis) versus temperature (*y*-axis), shows their liquidus curve as a characteristic W-shaped curve, suggesting the formation exclusively of a stable, congruently melting cocrystal phase.

This result confirms the formation of the (1:2) VLF-CAT cocrystal (0.33 molar fraction), where the melting temperature reaches its maximum (90 °C) among the studied compositions, suggesting the strongest interaction between the starting components.^3^ The TG-DTA curves show that the (1:2) VLF-CAT cocrystal is thermally stable up to 129 °C, with no significant mass loss observed below this temperature, indicating the absence of residual ethyl acetate. Above 129 °C, thermal decomposition occurs in three mass loss steps up to 571 °C (Figure S14).

Two eutectic points were identified at molar ratios close to 7:3 (molar fraction = 0.7) and 1:4 (0.2 molar fraction = 0.2), corresponding to the compositions at which the melting temperatures of the precursor-cocrystal mixture reach minima, giving rise to a characteristic V-shaped phase diagram.^5,54^ Furthermore, the asymmetric eutectic compositions observed at molar fractions of approximately 0.7 and 0.2 arise from the nonequimolar stoichiometry of the (1:2) VLF-CAT cocrystal and the distinct molecular features of venlafaxine and catechol. These compositions represent the lowest melting temperatures in the binary system, where the coexistence of the cocrystal phase with an excess of one precursor leads to melting point depression characteristic of eutectic mixtures.

PXRD patterns of the different molar fractions (Figure 6(a)) clearly indicate the formation of a (1:2) VLF-CAT cocrystal at the 0.33 molar fraction, as evidenced by the appearance of new diffraction peaks, consistent with the simulated PXRD pattern calculated from the structure solved from powder diffraction data, confirming the formation of the same crystalline phase (Figure 6(b)).

**Figure 6:**
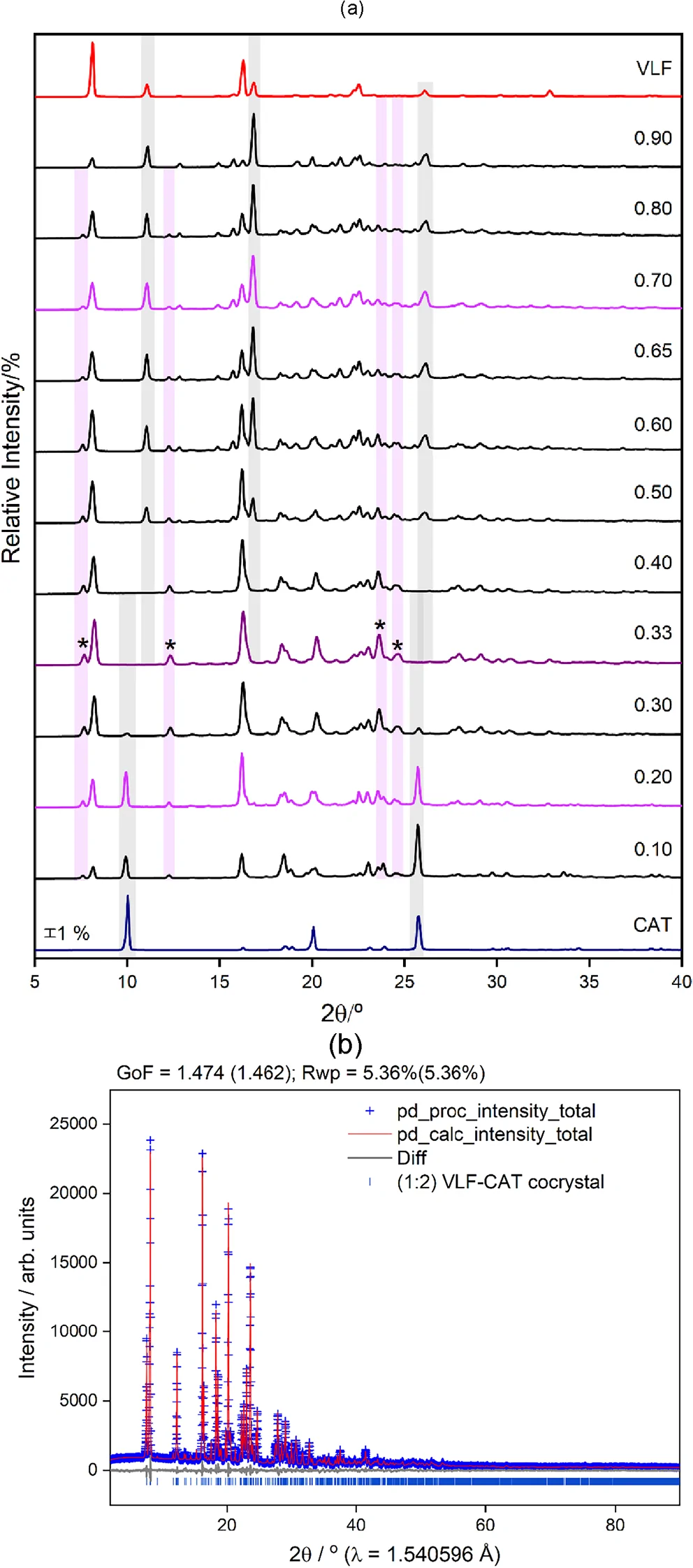
(a) PXRD pattern of VLF-CAT binary mixtures prepared at different molar fractions by mechanochemistry (LAG, 30 Hz and 30 min) and (b) PXRD pattern and results from Rietveld refinement.

The eutectic mixtures display reflections corresponding to both phases: VLF and the cocrystal at the 0.70 fraction, and CAT and the cocrystal at the 0.20 fraction, in agreement with the binary phase diagram. Table 3 summarizes the main diffraction peaks of VLF, the 0.70 eutectic mixture, the 0.33 cocrystal, the 0.20 eutectic mixture, and CAT.

**Table 3:** Main Diffraction Peaks of VLF, 0.70 Eutectic Mixture, 0.33 Cocrystal, 0.20 Eutectic Mixture, and CAT

Main peaks (2θ/°)											
**VLF**	/	8.13	/	11.06	/	16.29	16.85	/	/	/	26.10
**0.70**	7.60	8.13	/	11.06	12.31	16.22	16.82	23.56	24.60	/	26.11
**0.33**	7.67	8.20	/	/	12.32	16.29	/	23.63	24.68	/	/
**0.20**	7.58	8.15	9.93	/	12.27	16.21	/	23.61	24.55	25.73	/
**CAT**	/	/	10.04	/	/	16.26	/	/	/	25.72	/

The FTIR spectra of VLF, CAT, the eutectic mixtures, and the VLF-CAT cocrystal are shown in Figure 7. The FTIR spectrum of VLF shows a broad O–H stretching band at 3146 cm^–1^, a characteristic aromatic C=C stretching band at 1512 cm^–1^, a C–O–CH_3_ stretching band at 1239 cm^–1^, and a band at 1179 cm^–1^ attributed to C–N stretching vibrations.^55,56^ The FTIR spectrum of CAT exhibits two distinct O–H stretching bands at 3444 cm^–1^ and 3322 cm^–1^, attributed to free and hydrogen-bonded phenolic O–H groups, respectively.^27^ Characteristic aromatic C=C stretching vibrations are observed at 1618 and 1595 cm^–1^,^57^ while the band at 1466 cm^–1^ is assigned to C–O stretching of the phenolic group.^57^

**Figure 7:**
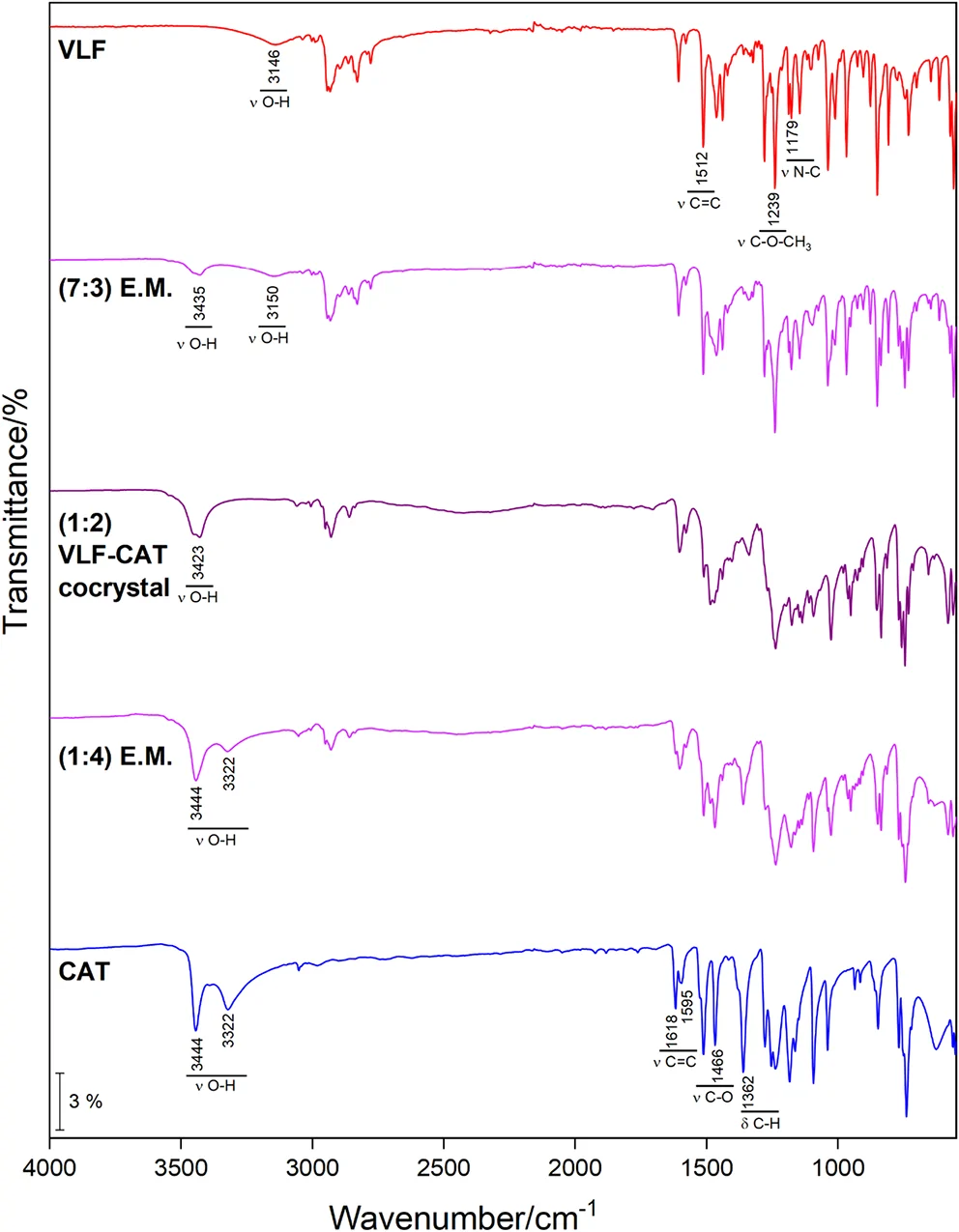
FTIR spectra of VLF, (7:3) eutectic mixture (E.M.), (1:2) VLF-CAT cocrystal, (1:4) eutectic mixture (E.M.), and CAT.

The FTIR spectra of both eutectic mixtures show the characteristic vibrational bands of their constituent phases, namely VLF and the cocrystal for the 7:3 composition, and the cocrystal and CAT for the 1:4 composition. These results confirm the coexistence of the individual crystalline phases without the formation of additional intermolecular interactions detectable by FTIR.

In contrast, the FTIR spectrum of the (1:2) VLF–CAT cocrystal exhibits significant spectral changes. The O–H stretching band of CAT shifts from 3444 to 3423 cm^–1^, indicating a change in the hydrogen-bonding environment upon cocrystal formation. This shift is accompanied by a decrease in the intensity of the O–H stretching band at 3322 cm^–1^. In addition, the band at 1362 cm^–1^, assigned to the aromatic C–H bending vibration of CAT, shows reduced intensity, whereas the band at 1145 cm^–1^, attributed to the C–N stretching vibration of VLF, increases in intensity. Furthermore, the O–H stretching band of VLF at 3146 cm^–1^ also exhibits reduced intensity after cocrystallization. These spectral changes provide further evidence for the formation of a new hydrogen-bonding network in the cocrystal. These intensity variations are consistent with changes in the local molecular environment arising from the new crystal packing and intermolecular interactions. Similar changes in infrared band intensities have been reported to accompany changes in hydrogen-bonding environments and should be interpreted alongside the observed wavenumber shifts rather than as independent evidence of hydrogen-bond formation.^58–60^ Overall, the combined band shifts and intensity variations are fully consistent with the hydrogen-bonding network revealed by the crystal structure of the (1:2) VLF–CAT cocrystal.

#### 3.5 Solubility Studies

The objective of this study was to investigate the effects of solid-state modification on the solubility of Venlafaxine. In addition to evaluating the solubility of the (1:2) VLF-CAT cocrystal, the pharmaceutical relevance of the eutectic mixtures was also investigated. Therefore, the apparent solubility of VLF and the newly obtained solid forms (eutectic mixtures and cocrystals) was evaluated using the shake-flask method followed by UV–Vis spectrophotometric quantification. The VLF, (1:2) VLF-CAT cocrystal, eutectic mixtures concentration (Table 4) was evaluated.

**Table 4:** Solubility Data of VLF and the Multicomponent Solids Obtained

	Concentration/mg mL^–1^	SD	RSD/%	Ratio system/API
Venlafaxine	0.248	4.80 × 10^–5^	0.48	-
(7:3) E.M.	14.98	0.47	3.15	60.3
(1:2) VLF-CAT cocrystal	18.07	0.40	2.20	72.7
(1:4) E.M.	17.84	0.40	2.22	71.8

The column plot of VLF concentrations for the (7:3) E.M., the (1:2) VLF-CAT cocrystal, and the (1:4) E.M. (Figure 8) clearly demonstrates the significant potential of multicomponent solid forms to enhance the apparent solubility of venlafaxine.

**Figure 8:**
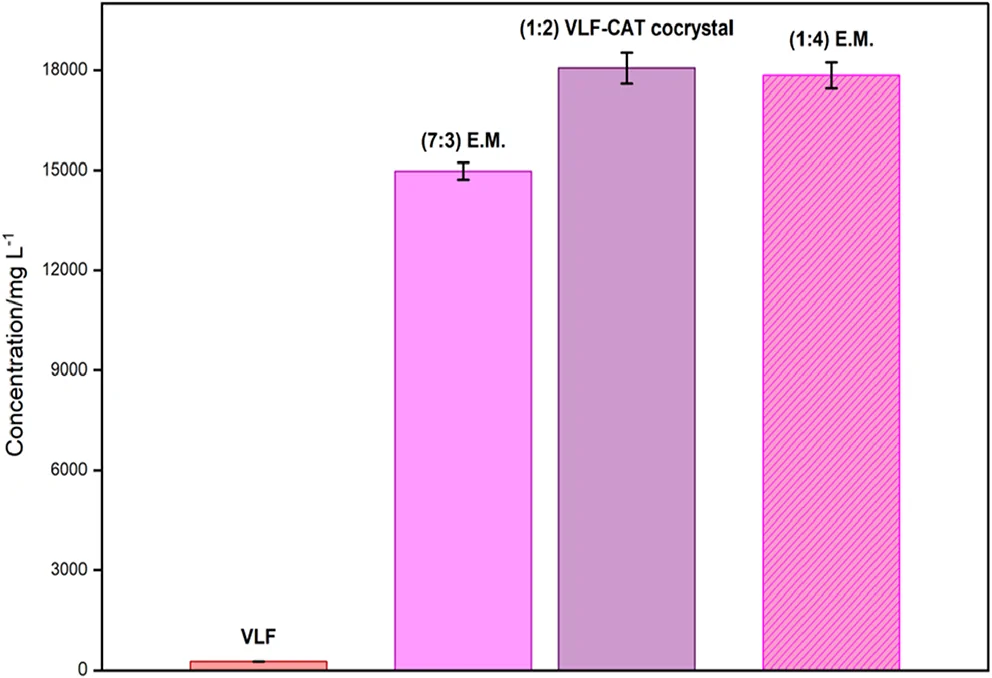
Column plot of VLF concentrations in the (7:3) eutectic mixture, (1:2) VLF-CAT cocrystal, and (1:4) eutectic mixture.

Previous studies reported a venlafaxine solubility of 0.39 mg mL^–1^, which is consistent with the value obtained in the present study (Table 4).^18^ The (1:2) VLF-CAT cocrystal exhibited the highest concentration (18.07 mg mL^–1^) in solution when compared to the eutectic mixtures. In pharmaceutical cocrystal, a homogeneous crystalline phase is formed through a new network of intermolecular interactions, which may reduce the crystal lattice energy relative to the parent crystalline components and thereby enhance solubility.^61,62^ In contrast, during the formation of eutectic mixtures, the starting compounds retain their individual crystalline phases, with the system being predominantly stabilized by weak adhesive-type interactions.

Additionally, the results demonstrate that eutectic mixtures also promote a considerable increase in the apparent solubility of venlafaxine compared to the pure drug. Several studies in the literature report the use of eutectic mixtures as an effective strategy to enhance the dissolution rate of pharmaceutical compounds.^48^

PXRD analysis of the solid residues recovered after the solubility assays (Figure S15) revealed that the precipitated (1:2) VLF-CAT cocrystal remained structurally unchanged, confirming the absence of any solid-state transformation during the experiment.

The (7:3) E.M. precipitate consisted exclusively of pure VLF. This behavior suggests that the high solubility of the cocrystal, combined with the less organized lattice arrangement characteristic of the eutectic phase, may have favored the solubilization of venlafaxine in solution.^48^ As a result, a concentration of 14.98 mg mL^–1^ was observed after 48 h of the experiment.

In comparison, the solid residue of the (1:4) E.M. system exhibited a diffraction pattern characteristic of the cocrystal (Figure S15), indicating that, at this drug-coformer ratio, there was an excess of solid cocrystal at the end of the experiment. In this context, these data indicate that the coformer that constituted the eutectic phase was completely solubilized in the medium. In contrast, part of the cocrystal remained as a solid phase and may even have been recrystallized during the experiment. This condition influenced the solid-solution equilibrium, resulting in a concentration of 17.84 mg mL^–1^ after 48 h.

These results suggest that, in both E.M. systems, the apparent drug concentration in solution is governed by the solubility of the cocrystal present in the composition.

To further investigate the solubilization mechanism, control experiments were performed using physical mixtures at the same VLF:CAT molar proportions as the eutectic mixtures. The 7:3 and 1:4 physical mixtures showed 28- and 33-fold higher VLF solubility than pure VLF, respectively, demonstrating that CAT contributes to VLF solubilization. However, these values remained lower than those obtained for the corresponding eutectic mixtures, indicating that the enhanced solubilization cannot be attributed solely to the presence of CAT. PXRD analysis of the recovered solid residues showed that the residues from the physical mixtures were consistent with those obtained from the corresponding eutectic mixtures (Figure S16), indicating that similar solid-state transformations occur upon contact with the aqueous medium. In both physical mixtures, CAT was completely dissolved, whereas only a fraction of VLF was dissolved. The dissolved CAT may therefore promote the in situ formation of the (1:2) VLF-CAT cocrystal upon contact with the remaining VLF-rich solid phase, contributing to VLF solubilization. The lower solubility observed for the physical mixtures compared with the corresponding eutectic mixtures may be related to differences in the extent of cocrystal formation and to the high concentration of dissolved CAT in the aqueous phase, which may limit further dissolution of the cocrystal. Overall, these results indicate that the enhanced solubilization of the eutectic mixtures likely arises from the combined effects of CAT-mediated solubilization and solid-state transformation, including (1:2) VLF-CAT cocrystal formation.

## 4 Conclusion

In this work, a comprehensive investigation of venlafaxine and catechol multicomponent solid forms was conducted, including the identification and characterization of a novel cocrystal and eutectic mixtures. The combination of solid-state analyses, phase diagram studies, and solubility assessments provided a thorough understanding of the structural and physicochemical differences among the compounds obtained. The results demonstrate that cocrystallization and eutectic formation are effective strategies to modulate the solubility and stability of poorly water-soluble drugs. Overall, this work provides a valuable framework for the rational design of multicomponent solid forms and highlights the potential of these approaches to improve the performance and therapeutic applicability of venlafaxine and similar APIs.

## Supplementary Material



## Notes

Additional PXRD patterns, long-term stability of the (1:2) VLF-CAT cocrystal, crystallographic tables (atomic coordinates, bond lengths, bond angles, and hydrogen atom coordinates), UV–Vis selectivity analysis, calibration data, analytical curves, and quantification of the cocrystal and eutectic mixture data, optical microscopy images and video of the VLF-CAT physical mixture, TG-DTA curve, and PXRD patterns of solid residues after solubility studies (PDF)
