# Supplementary material for: Solid-State Investigation of a Novel (1:2) Venlafaxine-Catechol Cocrystal: Structural, Thermal, Spectroscopic, and Solubility Studies
Source: ACS Omega. 2026 Oct 8;11(41):62777–88. doi: 10.1021/acsomega.6c04465 (PMC13689160; doi:10.1021/acsomega.6c04465)
Supplement: Supplementary file 1 [file ao6c04465_si_001.pdf]

Supplementary Information

Solid-state investigation of a novel (1:2) venlafaxine-catechol cocrystal: Structural, thermal, spectroscopic and solubility studies

Giovanna de Paula Costa[1], Patrícia Osório Ferreira[2], Guilherme Isquibola[3], Juliana Guedes dos Santos[1], Fabio Furlan Ferreira[2], Flávio Junior Caires[1*]

[1]School of Sciences, São Paulo State University, Bauru, 17033-360, SP, Brazil

[2]LCCEM / CCNH, Federal University of ABC, Santo Andre, 09280-560, SP, Brazil

[3]Institute of Chemistry, São Paulo State University, Araraquara, 14800-900, SP, Brazil

Corresponding author:

Flávio J. Caires

São Paulo State University (UNESP), School of Sciences, Department of Chemistry

Av. Eng. Luís Edmundo Carrijo Coube, 2085 - Nucleo Res. Pres. Geisel,17033-360, Bauru, Brazil.

E-mail: flavio.caires@unesp.br

Tel: +55 1431036088

Fig. S1. PXRD pattern of VLF free base obtained and simulated for VLF form I [1].

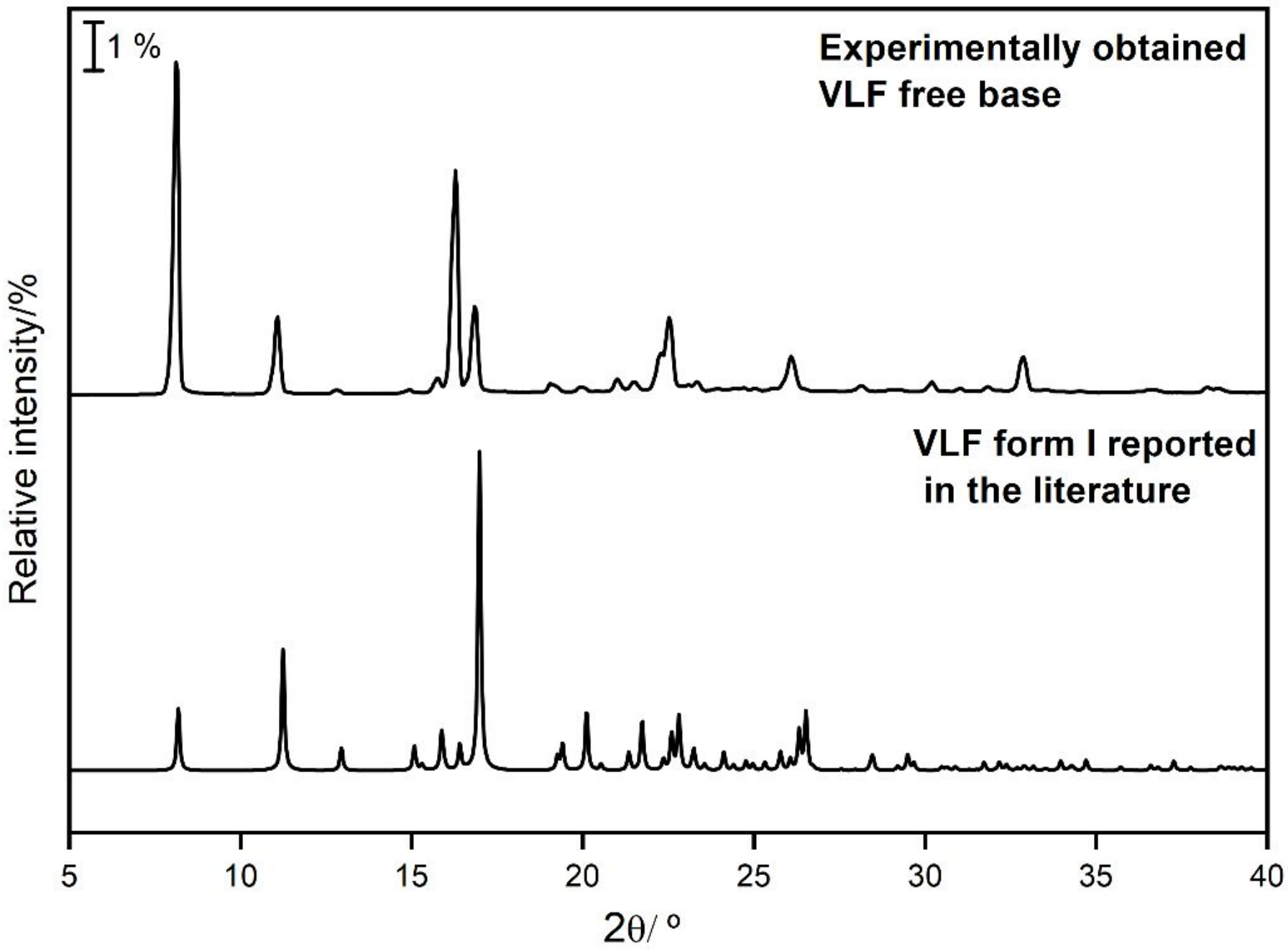

Fig. S2. PXRD pattern of (1:2) VLF-CAT after 12 months of storage in a desiccator, indicating its structural stability.

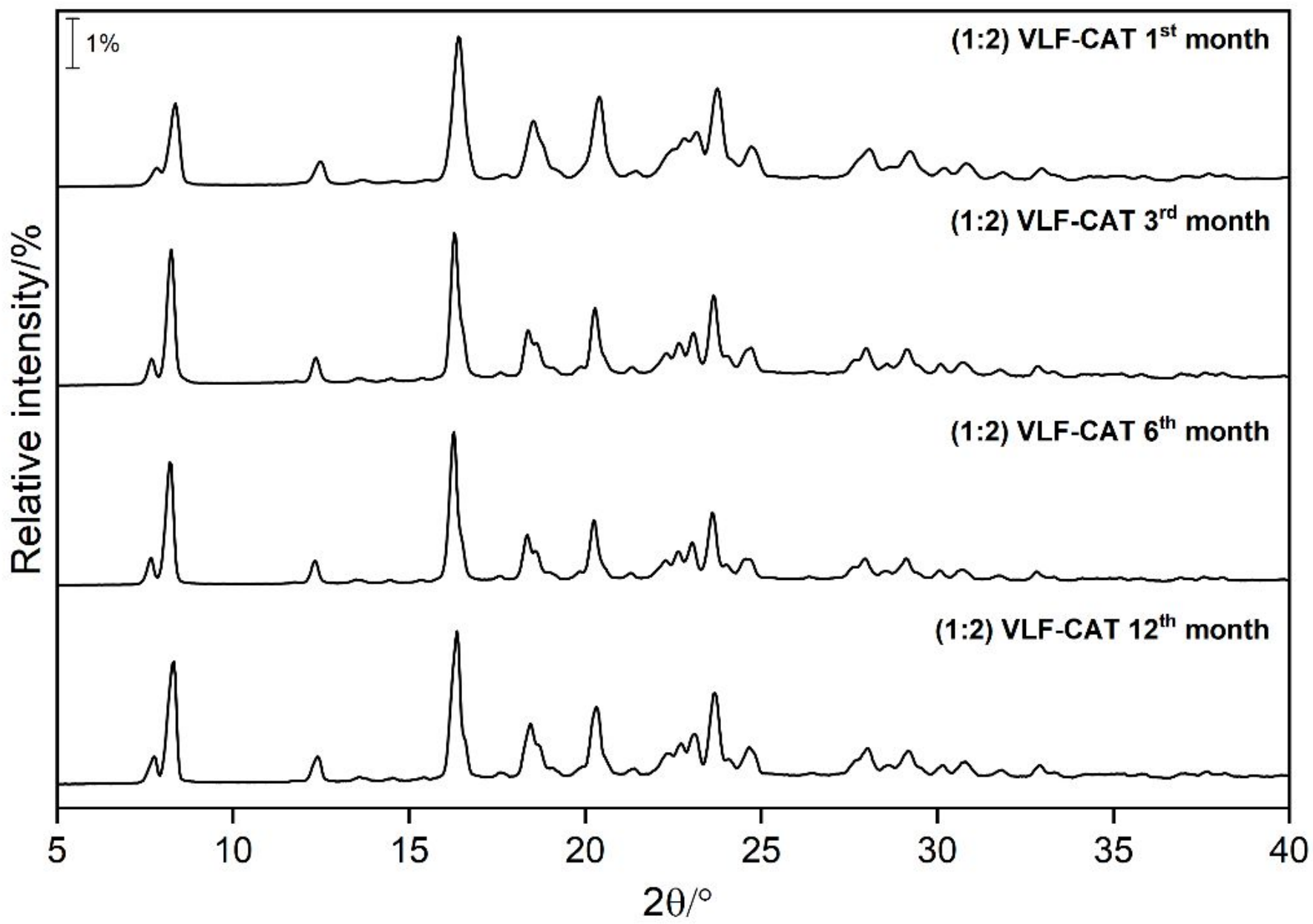

Table S3. Fractional Atomic Coordinates (x10^4) and Equivalent Isotropic Displacement Parameters (Å^{2}x10^3) for VLF-CAT. U_{eq} is defined as 1/3 of the trace of the orthogonalised U_{ij}.

Atom	x	y	z	U_{eq}
C1	4360(40)	2830(40)	1410(30)	54.8
C2	3500(40)	2070(40)	240(30)	54.8
C3	4500(30)	2190(40)	2190(30)	54.8
C4	3860(40)	4120(40)	2060(30)	54.8

O5	4160(20)	950(20)	-415(17)	54.8
C6	2010(30)	1790(30)	400(30)	54.8
C7	3530(30)	2750(40)	-500(30)	54.8
N8	5630(20)	2740(30)	3120(20)	54.8
C9	4610(30)	5060(40)	2030(30)	54.8
C10	2630(30)	4430(40)	2660(40)	54.8
C11	1110(30)	1200(40)	-740(30)	54.8
C12	2770(30)	2040(40)	-1660(20)	54.8
C13	6030(30)	1960(30)	3610(30)	54.8
C14	5260(30)	3910(30)	4060(30)	54.8
C15	4180(30)	6240(40)	2570(30)	54.8
C16	2190(30)	5620(40)	3220(30)	54.8
C17	1230(30)	1860(30)	-1480(30)	54.8
C18	2980(40)	6530(40)	3170(40)	54.8
O19	2700(20)	7730(30)	3660(20)	54.8
C20	1470(20)	8100(30)	4280(20)	54.8
C21	8160(40)	2990(40)	1170(40)	54.8
C22	7660(30)	2250(50)	30(40)	54.8
C23	8710(30)	4170(40)	1500(30)	54.8
O24	8100(20)	2560(20)	1930(20)	54.8
C25	7710(30)	2690(40)	-800(30)	54.8
O26	7150(20)	1120(20)	-260(18)	54.8
C27	8750(30)	4610(40)	690(30)	54.8
C28	8260(30)	3880(40)	-460(30)	54.8
C29	1650(40)	3040(40)	4820(30)	54.8
C30	740(40)	2640(40)	3820(30)	54.8
C31	2530(30)	2220(40)	4980(30)	54.8
O32	1710(20)	4240(30)	5685(17)	54.8
C33	710(30)	1420(40)	2980(40)	54.8
O34	-80(20)	3470(20)	3721(15)	54.8
C35	2490(30)	1020(40)	4130(30)	54.8
C36	1600(30)	620(40)	3160(30)	54.8

Table S4. Bond Lengths in Å for VLF-CAT.

Atom	Atom	Length
C1	C2	1.57(5)
C1	C3	1.54(7)
C1	C4	1.51(6)
C1	H1	0.96(6)
C2	O5	1.41(4)
C2	C6	1.51(5)
C2	C7	1.54(8)
C3	N8	1.49(4)
C3	H2	0.96(6)
C3	H3	0.96(5)
C4	C9	1.37(7)
C4	C10	1.39(5)
O5	H4	0.99(3)
C6	C11	1.54(5)

C6	H5	0.96(4)
C6	H6	0.96(6)
C7	C12	1.51(4)
C7	H7	0.95(4)
C7	H8	0.96(5)
N8	C13	1.42(6)
N8	C14	1.46(4)
C9	C15	1.36(6)
C9	H9	0.96(4)
C10	C16	1.38(6)
C10	H10	0.95(7)
C11	C17	1.52(7)
C11	H11	0.95(4)
C11	H12	0.96(6)
C12	C17	1.53(4)
C12	H13	0.96(5)
C12	H14	0.96(7)
C13	H15	0.96(4)
C13	H16	0.96(4)
C13	H17	0.96(5)
C14	H18	0.96(4)
C14	H19	0.96(5)
C14	H20	0.96(6)
C15	C18	1.37(5)
C15	H21	0.96(7)
C16	C18	1.37(7)
C16	H22	0.96(4)
C17	H23	0.96(4)
C17	H24	0.96(4)
C18	O19	1.34(5)
O19	C20	1.41(3)
C20	H25	0.96(5)
C20	H26	0.97(5)
C20	H27	0.96(3)
C21	C22	1.40(6)
C21	C23	1.40(7)
C21	O24	1.32(7)
C22	C25	1.41(8)
C22	O26	1.34(6)
C23	C27	1.39(7)
C23	H28	0.96(4)
O24	H29	0.99(3)
C25	C28	1.41(6)
C25	H30	0.96(4)
O26	H31	0.99(4)
C27	C28	1.41(4)
C27	H32	0.96(6)
C28	H33	0.97(7)
C29	C30	1.42(5)
C29	C31	1.40(7)

C29	O32	1.38(4)
C30	C33	1.39(5)
C30	O34	1.34(5)
C31	C35	1.38(5)
C31	H34	0.96(5)
O32	H35	0.99(5)
C33	C36	1.39(7)
C33	H36	0.95(6)
O34	H37	0.99(3)
C35	C36	1.38(5)
C35	H38	0.96(7)
C36	H39	0.96(5)

Table S5. Bond Angles in ° for VLF-CAT.

Atom	Atom	Atom	Angle/°
C2	C1	C3	114(3)
C2	C1	C4	113(3)
C2	C1	H1	106(4)
C3	C1	C4	112(3)
C3	C1	H1	105(4)
C4	C1	H1	106(4)
C1	C2	O5	110(3)
C1	C2	C6	113(3)
C1	C2	C7	110(3)
O5	C2	C6	108(3)
O5	C2	C7	105(3)
C6	C2	C7	110(3)
C1	C3	N8	113(3)
C1	C3	H2	109(4)
C1	C3	H3	109(4)
N8	C3	H2	113(4)
N8	C3	H3	104(4)
H2	C3	H3	109(5)
C1	C4	C9	119(4)
C1	C4	C10	124(4)
C9	C4	C10	117(4)
C2	O5	H4	108(3)
C2	C6	C11	113(3)
C2	C6	H5	110(4)
C2	C6	H6	108(4)
C11	C6	H5	108(4)
C11	C6	H6	108(4)
H5	C6	H6	109(4)
C2	C7	C12	112(3)
C2	C7	H7	107(4)
C2	C7	H8	108(4)
C12	C7	H7	109(4)
C12	C7	H8	110(4)
H7	C7	H8	110(5)

C3	N8	C13	113(3)
C3	N8	C14	113(3)
C13	N8	C14	108(3)
C4	C9	C15	122(4)
C4	C9	H9	118(4)
C15	C9	H9	120(4)
C4	C10	C16	122(4)
C4	C10	H10	119(5)
C16	C10	H10	118(5)
C6	C11	C17	114(3)
C6	C11	H11	109(4)
C6	C11	H12	108(4)
C17	C11	H11	109(4)
C17	C11	H12	107(4)
H11	C11	H12	110(5)
C7	C12	C17	109(3)
C7	C12	H13	111(4)
C7	C12	H14	108(4)
C17	C12	H13	112(4)
C17	C12	H14	107(4)
H13	C12	H14	109(4)
N8	C13	H15	108(4)
N8	C13	H16	114(4)
N8	C13	H17	107(4)
H15	C13	H16	109(4)
H15	C13	H17	110(4)
H16	C13	H17	109(4)
N8	C14	H18	104(4)
N8	C14	H19	111(4)
N8	C14	H20	113(4)
H18	C14	H19	109(4)
H18	C14	H20	110(4)
H19	C14	H20	110(4)
C9	C15	C18	121(4)
C9	C15	H21	120(4)
C18	C15	H21	119(5)
C10	C16	C18	119(4)
C10	C16	H22	121(5)
C18	C16	H22	120(5)
C11	C17	C12	110(3)
C11	C17	H23	109(4)
C11	C17	H24	108(4)
C12	C17	H23	108(4)
C12	C17	H24	111(4)
H23	C17	H24	110(5)
C15	C18	C16	120(4)
C15	C18	O19	114(4)
C16	C18	O19	126(4)
C18	O19	C20	117(3)
O19	C20	H25	109(3)

O19	C20	H26	110(3)
O19	C20	H27	110(3)
H25	C20	H26	109(4)
H25	C20	H27	110(4)
H26	C20	H27	109(4)
C22	C21	C23	121(4)
C22	C21	O24	119(4)
C23	C21	O24	120(4)
C21	C22	C25	120(4)
C21	C22	O26	119(4)
C25	C22	O26	121(4)
C21	C23	C27	120(4)
C21	C23	H28	120(5)
C27	C23	H28	120(4)
C21	O24	H29	111(3)
C22	C25	C28	119(4)
C22	C25	H30	120(5)
C28	C25	H30	121(4)
C22	O26	H31	122(3)
C23	C27	C28	121(4)
C23	C27	H32	119(4)
C28	C27	H32	120(4)
C25	C28	C27	120(4)
C25	C28	H33	120(4)
C27	C28	H33	120(4)
C30	C29	C31	121(4)
C30	C29	O32	123(4)
C31	C29	O32	117(4)
C29	C30	C33	120(4)
C29	C30	O34	118(4)
C33	C30	O34	122(4)
C29	C31	C35	118(4)
C29	C31	H34	121(4)
C35	C31	H34	121(4)
C29	O32	H35	121(3)
C30	C33	C36	118(4)
C30	C33	H36	121(5)
C36	C33	H36	121(5)
C30	O34	H37	121(3)
C31	C35	C36	122(4)
C31	C35	H38	119(4)
C36	C35	H38	119(4)
C33	C36	C35	121(4)
C33	C36	H39	119(4)
C35	C36	H39	120(4)

Table S6. Hydrogen Fractional Atomic Coordinates ($x10^4$) and Equivalent Isotropic Displacement Parameters ($Å^2x10^3$) for VLF-CAT. U_{eq} is defined as 1/3 of the trace of the orthogonalised U_{ij}.

Atom	x	y	z	U_{eq}
H1	5300(40)	2900(40)	1210(30)	65.8
H2	4610(30)	1330(40)	1710(30)	65.8
H3	3660(30)	2310(40)	2600(30)	65.8
H4	5190(20)	1120(20)	-324(17)	65.8
H5	1570(30)	2530(30)	920(30)	65.8
H6	2030(30)	1230(30)	730(30)	65.8
H7	4490(30)	2870(40)	-610(30)	65.8
H8	3100(30)	3530(40)	-80(30)	65.8
H9	5450(30)	4870(40)	1610(30)	65.8
H10	2070(30)	3800(40)	2690(40)	65.8
H11	150(30)	1180(40)	-570(30)	65.8
H12	1420(30)	380(40)	-1190(30)	65.8
H13	3210(30)	1280(40)	-2110(20)	65.8
H14	2800(30)	2530(40)	-2060(20)	65.8
H15	6250(30)	1180(30)	2990(30)	65.8
H16	6820(30)	2270(30)	4160(30)	65.8
H17	5230(30)	1870(30)	3990(30)	65.8
H18	6080(30)	4150(30)	4580(30)	65.8
H19	4470(30)	3810(30)	4450(30)	65.8
H20	5070(30)	4520(30)	3810(30)	65.8
H21	4740(30)	6880(40)	2550(30)	65.8
H22	1340(30)	5820(40)	3630(30)	65.8
H23	770(30)	1370(30)	-2220(30)	65.8
H24	780(30)	2630(30)	-1100(30)	65.8
H25	1400(20)	8970(30)	4590(20)	65.8
H26	1510(20)	7890(30)	4910(20)	65.8
H27	660(20)	7700(30)	3780(20)	65.8
H28	9060(30)	4670(40)	2280(30)	65.8
H29	7140(20)	2620(20)	2210(20)	65.8
H30	7360(30)	2190(40)	-1580(30)	65.8
H31	7110(20)	790(20)	302(18)	65.8
H32	9120(30)	5420(40)	920(30)	65.8
H33	8290(30)	4190(40)	-1020(30)	65.8
H34	3150(30)	2490(40)	5660(30)	65.8
H35	1110(20)	4870(30)	5633(17)	65.8
H36	90(30)	1140(40)	2310(40)	65.8
H37	-740(20)	3240(20)	3040(15)	65.8
H38	3080(30)	440(40)	4230(30)	65.8
H39	1600(30)	-220(40)	2590(30)	65.8

Fig. S7. Selectivity analysis of Venlafaxine, Catechol, 0.33 VLF-CAT cocrystal, 0.7 VLF-CAT E.M., and 0.2 VLF-CAT E.M.

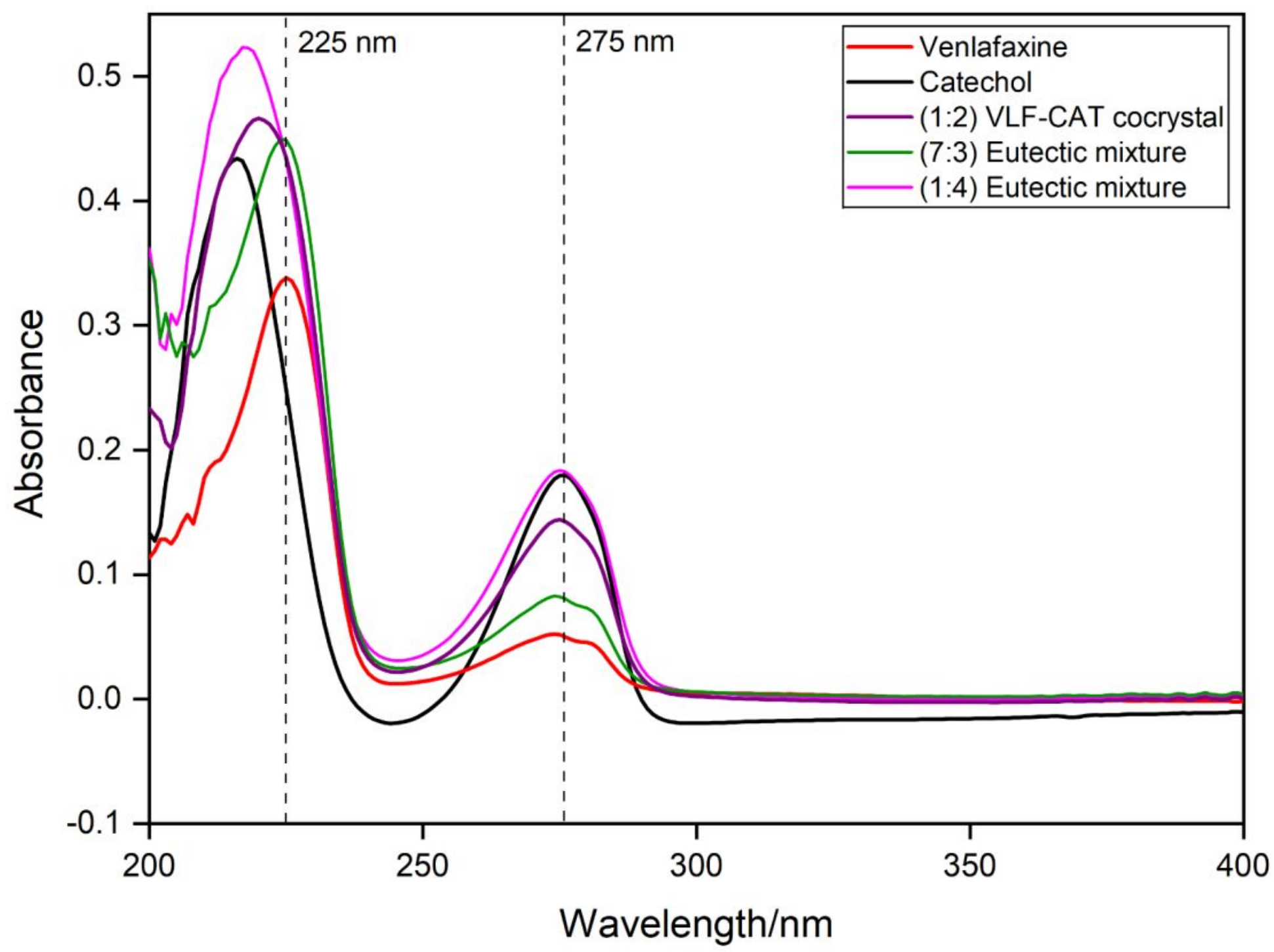

Drug quantification was carried out via a mathematical deconvolution approach based on simultaneous equations derived from the Beer–Lambert law and applied to the spectrophotometric signals [2]. Using Equations 1 and 2, two matrices (2×2 and 1×1) were constructed, and the drug concentration was obtained by multiplying the inverse of the 2×2 matrix by the 1×1 matrix.

$$A^{\lambda_1} = \varepsilon_1^{\lambda_1}.b.C_1 + \varepsilon_2^{\lambda_1}.b.C_2 \text{ (Equation 1)}$$

$$A^{\lambda_2} = \varepsilon_1^{\lambda_2}.b.C_1 + \varepsilon_2^{\lambda_2}.b.C_2 \text{ (Equation 2)}$$

Where,

A^{λ_1} represents the absorbance of the mixture at the maximum wavelength of the drug;

A^{λ_2} represents the absorbance of the mixture at the maximum wavelength of the coformer;

$\varepsilon_1^{\lambda_1}$ represents the molar absorptivity, determined from the slope of the analytical curve of the drug at its maximum absorption wavelength;

$\varepsilon_2^{\lambda_1}$ represents the molar absorptivity, determined from the slope of the analytical curve of the coformer at the maximum absorption wavelength of the drug;

$\varepsilon_1^{\lambda_2}$ represents the molar absorptivity, determined from the slope of the analytical curve of the drug at the maximum absorption wavelength of the coformer;

$\varepsilon_2^{\lambda_2}$ represents the molar absorptivity, determined from the slope of the analytical curve of the coformer at its maximum absorption wavelength;

b represents the optical path length;

C_1 represents the drug concentration;

C_2 represents the coformer concentration.

Table S8. Concentration and absorbance data from the analytical curve of VLF and CAT at 225 and 275 nm.

**VLF**	**Abs**		**CAT**	**Abs**	
Concentration (μg mL^{-1})	225 nm	275 nm	Concentration (μg mL^{-1})	225 nm	275 nm
5	0.21	0.03	8	0.20	0.14
7.5	0.31	0.04	12	0.30	0.20
10	0.41	0.05	16	0.42	0.29
12.5	0.50	0.06	20	0.53	0.37
15	0.60	0.07	24	0.63	0.44
20	0.80	0.10	28	0.73	0.52
-	-	-	36	0.94	0.67
-	-	-	44	1.13	0.82

Fig. S9. Analytical curves of (a) VLF e (b) CAT.

(a)

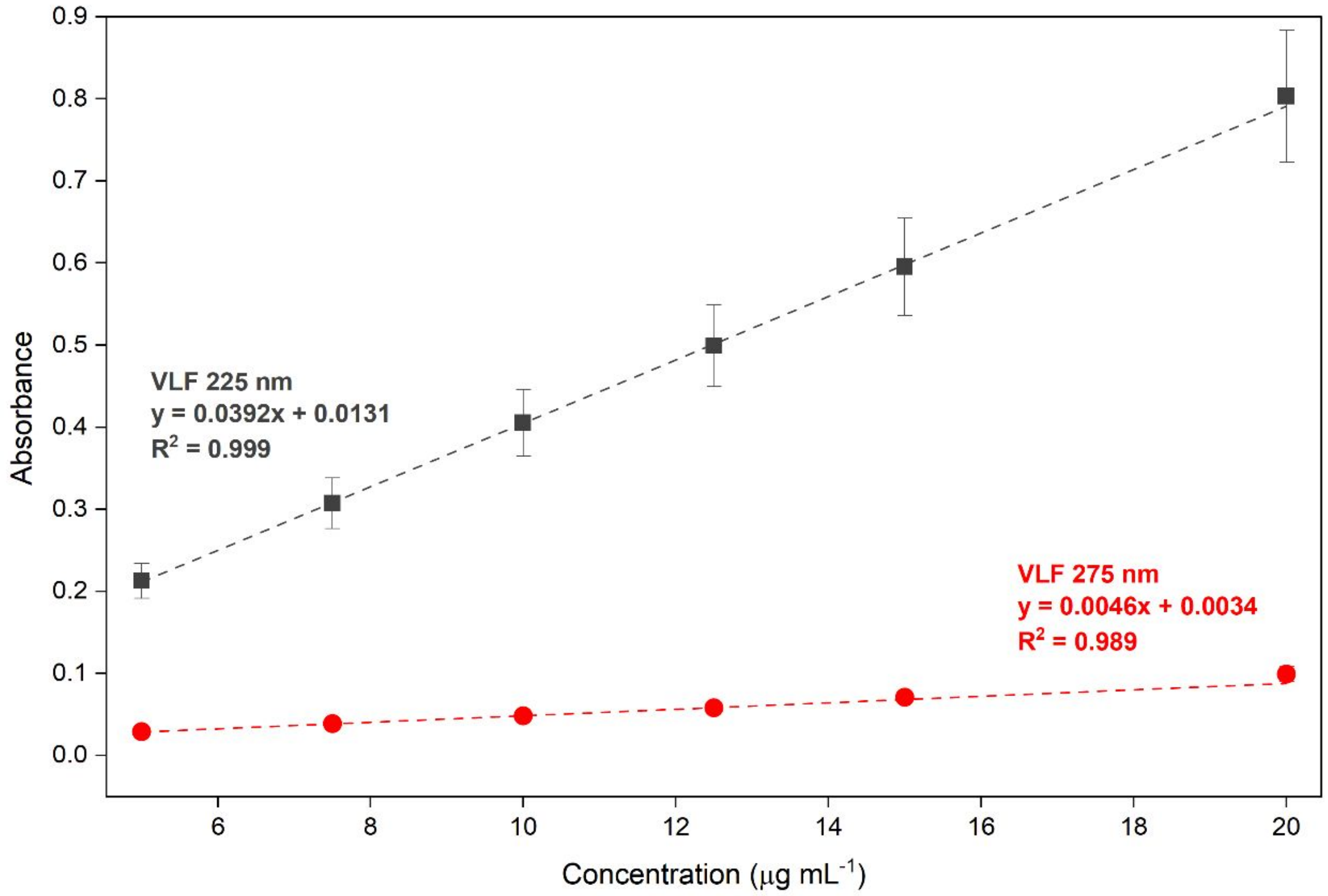

(b)

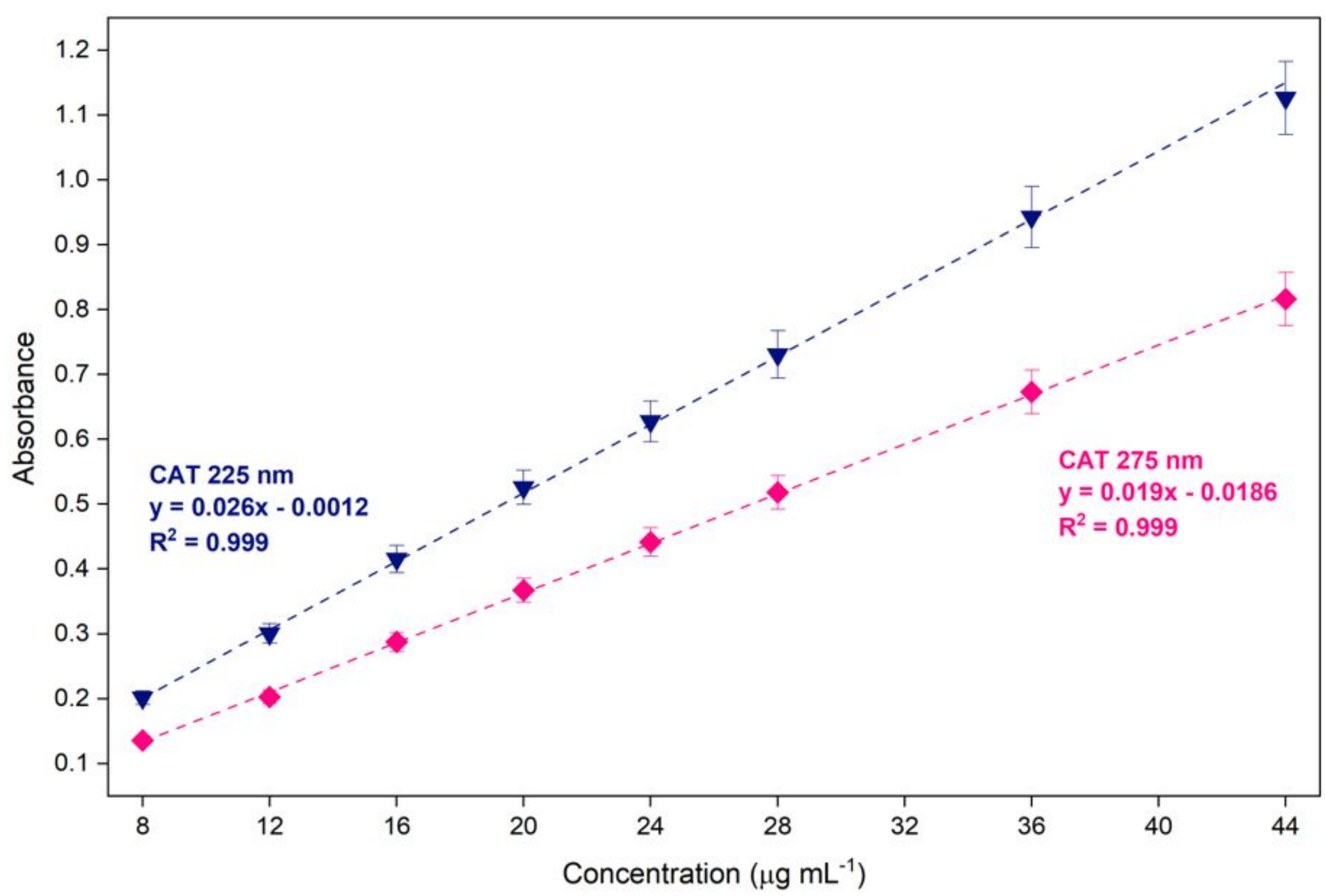

Table S10. Data from the quantification of the cocrystal (1:2) VLF-CAT, and in the eutectic mixtures (7:3) E.M. and (1:4) E.M.

(1:2) VLF-CAT cocrystal						
λ (nm)	*ε VLF*	*ε CAT*	*A*	**C (µg mL⁻¹)*		*C (µg mL⁻¹)*
225	0.03921	0.02596	0.52319	8.94101	CVLF	17882.01
275	0.00463	0.01910	0.16842	6.65018	CCAT	13300.36
225	0.03921	0.02596	0.53282	8.89626	CVLF	17792.52
275	0.00463	0.01910	0.17659	7.08877	CCAT	14177.53
225	0.03921	0.02596	0.54185	9.26059	CVLF	18521.18
275	0.00463	0.01910	0.17441	6.88632	CCAT	13772.63

(7:3) Eutectic mixture						
λ (nm)	*ε VLF*	*ε CAT*	*A*	**C (µg mL⁻¹)*		*C (µg mL⁻¹)*
225	0.03921	0.02596	0.47312	10.16210	CVLF	14517.27
275	0.00463	0.01910	0.10200	2.87677	CCAT	4109.67
225	0.03921	0.02596	0.49047	10.46376	CVLF	14948.23
275	0.00463	0.01910	0.10746	3.08950	CCAT	4413.57
225	0.03921	0.02596	0.49342	10.82257	CVLF	15460.80
275	0.00463	0.01910	0.10094	2.66117	CCAT	3801.67

(1:4) Eutectic mixture						
λ (nm)	*ε VLF*	*ε CAT*	*A*	**C (µg mL⁻¹)*		*C (µg mL⁻¹)*
225	0.03921	0.02596	0.53623	5.26721	CVLF	17557.37
275	0.00463	0.01910	0.26700	12.70188	CCAT	42339.60
225	0.03921	0.02596	0.52396	5.48855	CVLF	18295.18
275	0.00463	0.01910	0.25261	11.89484	CCAT	39649.46
225	0.03921	0.02596	0.52820	5.30194	CVLF	17673.13
275	0.00463	0.01910	0.26025	12.34007	CCAT	41133.55

**C represents the concentration of the compounds obtained from the matrix.*

Fig. S11. PXRD pattern comparative of compound by LAG and NG.

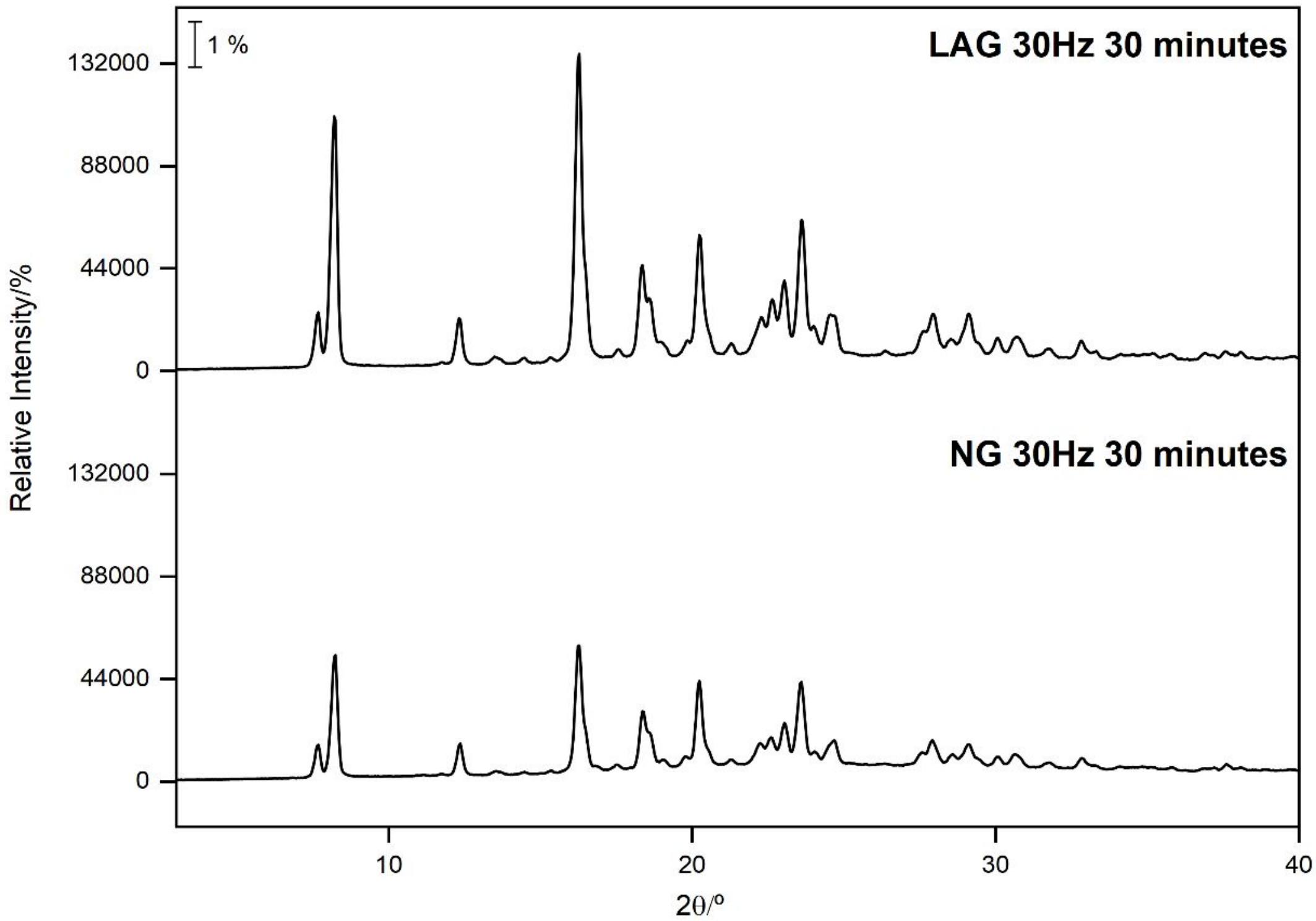

Fig. S12. Microscopy images of VLF-CAT physical mixture for 10 minutes.

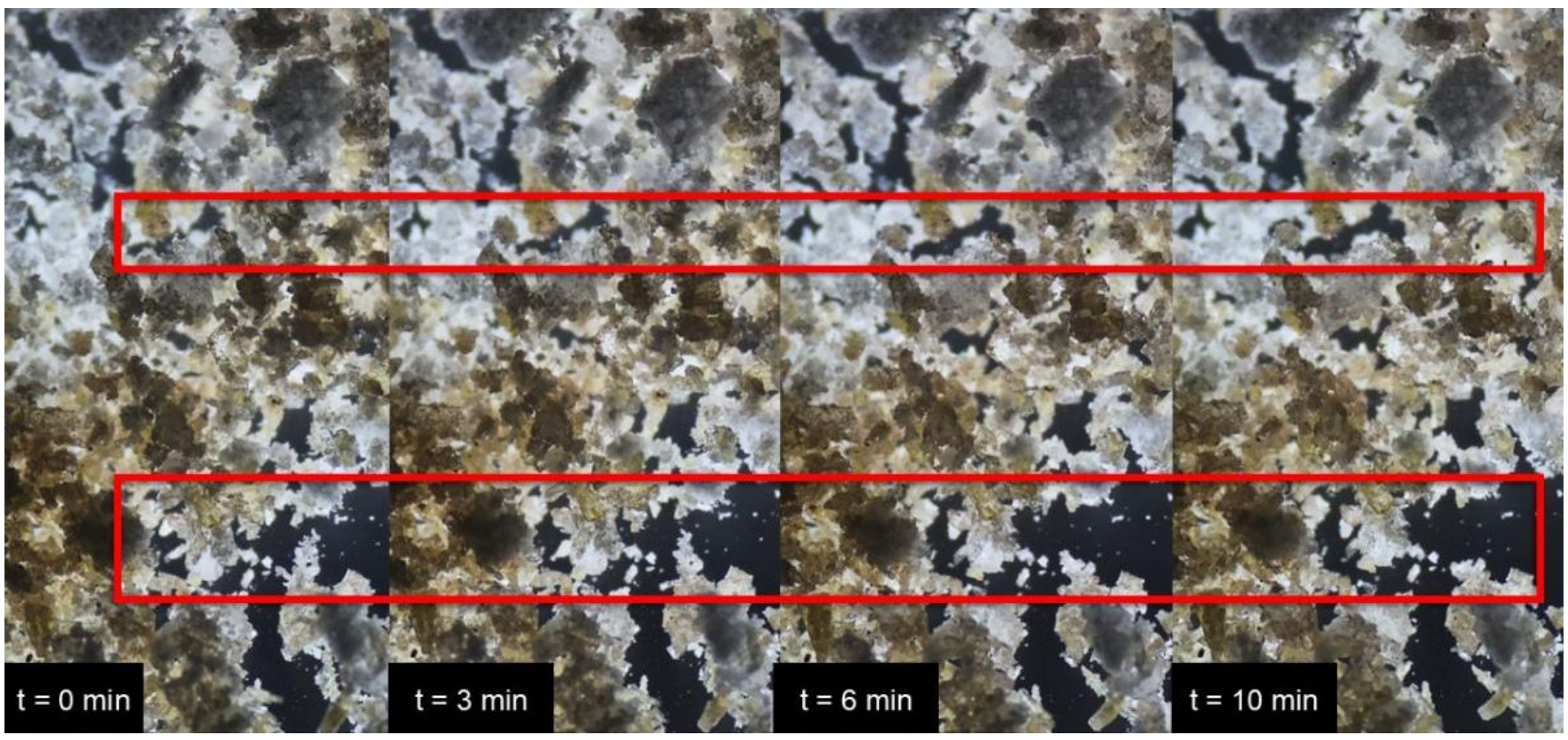

Video S13. Microscopy video of VLF-CAT physical mixture for 10 minutes accelerated 50x.

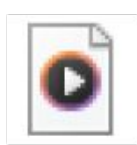

Video_S13_Microsc
opy_video_VLF-CAT_

Fig. S14. TG-DTA curve of (1:2) VLF-CAT.

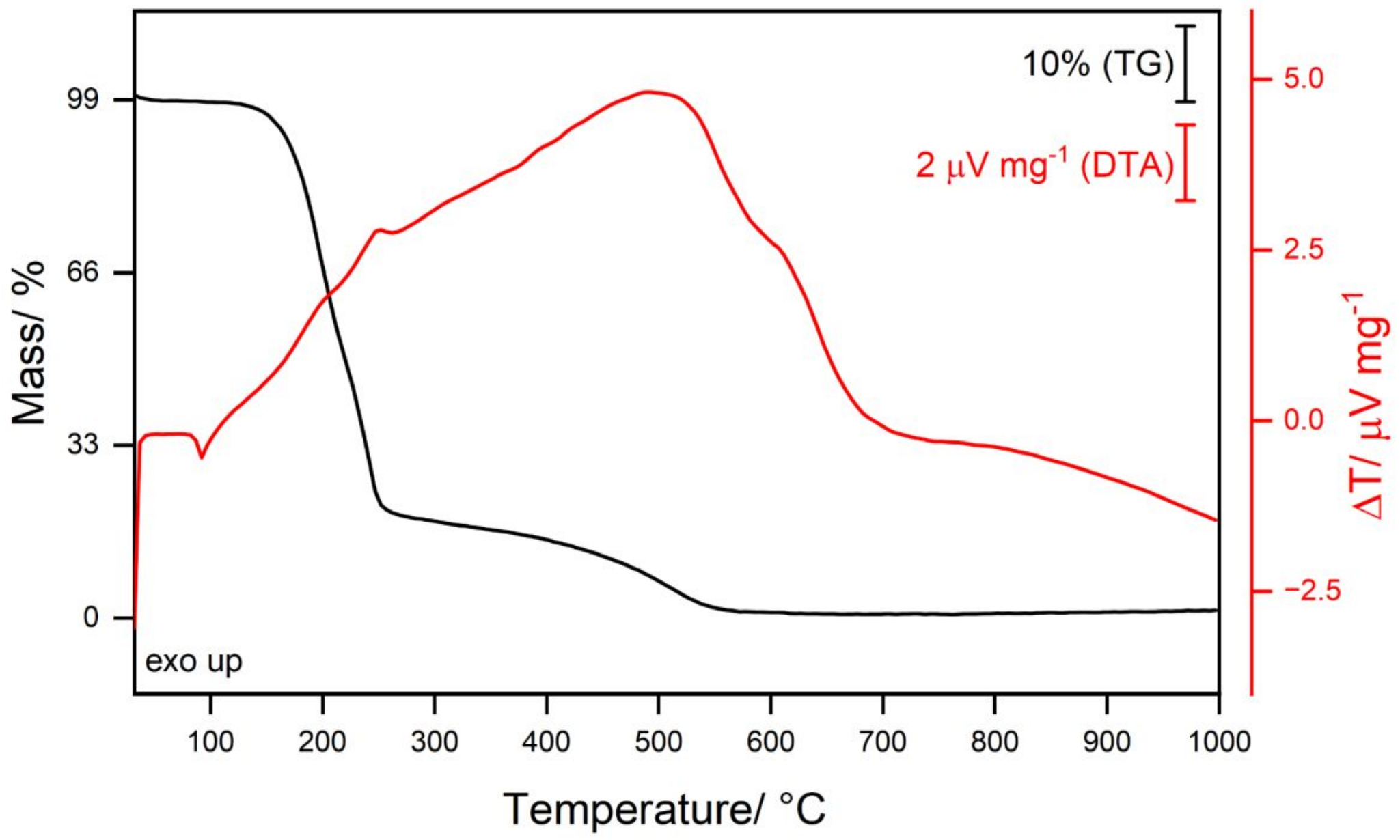

Fig. S15. PXRD patterns of the solid residues of the compounds before and after the solubility studies.

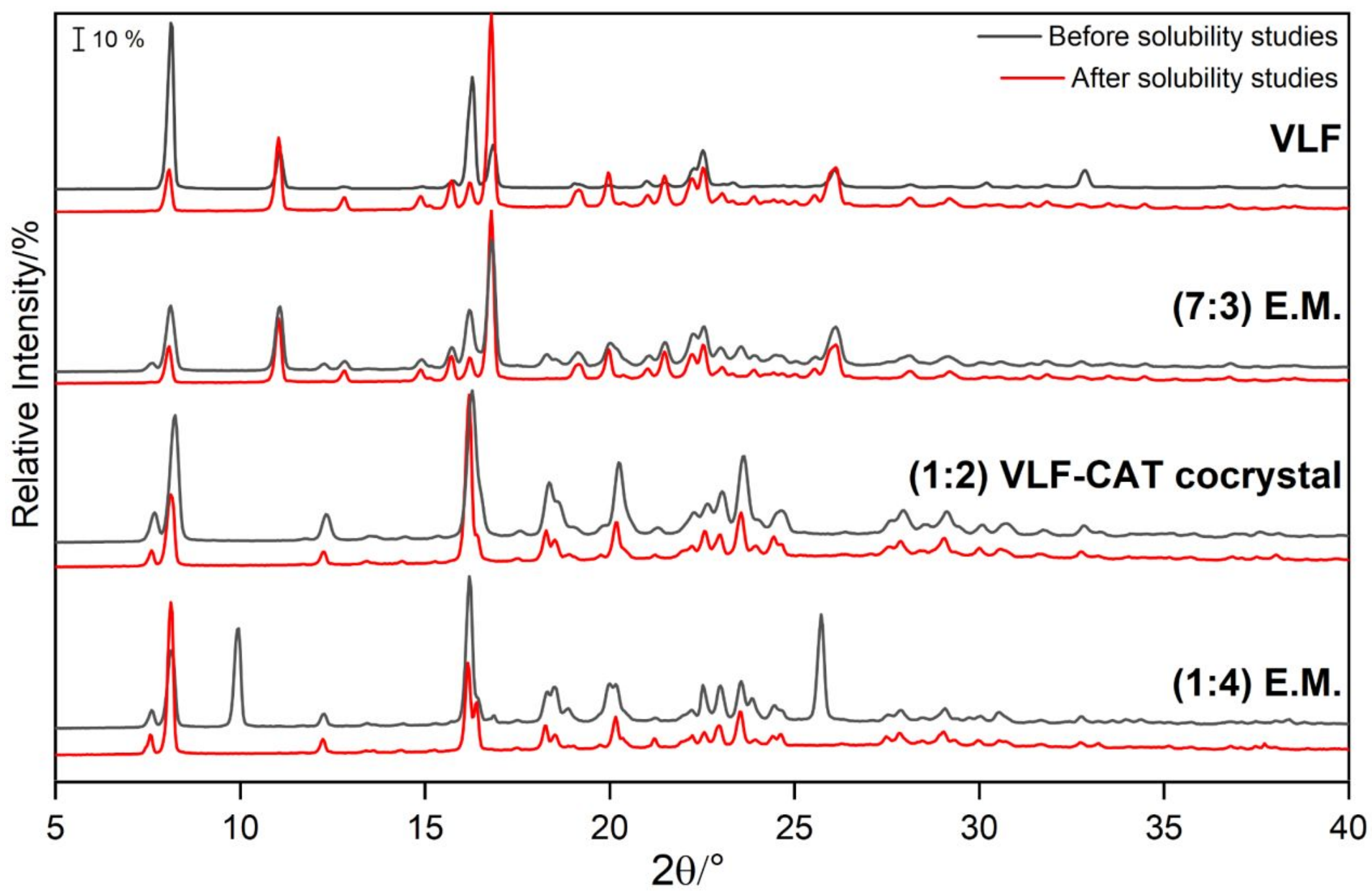

Fig. S16. PXRD patterns of the solid residues of physical mixtures and eutectic mixtures after solubility experiments.

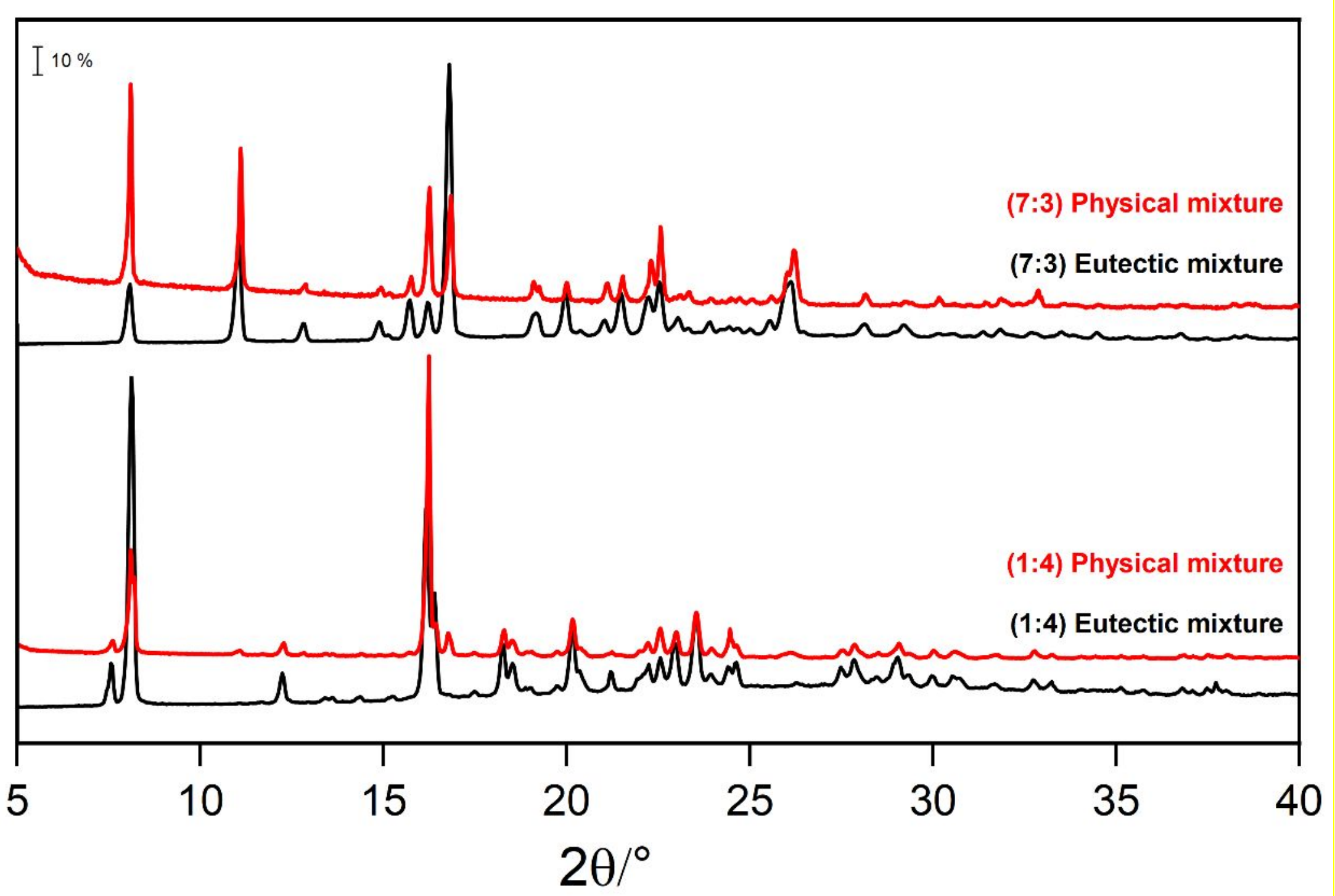

REFERENCES

[1] J.T.H. Van Eupen, W.W.J. Elffrink, R. Keltjens, P. Bennema, R. De Gelder, J.M.M. Smits, E.R.H. Van Eck, A.P.M. Kentgens, M.A. Deij, H. Meekes, E. Vlieg, Polymorphism and migratory chiral resolution of the free base of venlafaxine. A remarkable topotactical solid state transition from a racemate to a racemic conglomerate, Cryst. Growth Des. 8 (2008) 71–79. https://doi.org/10.1021/cg700831z.

[2] L.T. Ferreira, Sistemas farmacêuticos multicomponentes : sais e sistemas eutéticos profundos terapêuticos (THEDES) dos insumos farmacêuticos ativos genfibrozila , cetoprofeno e candesartana, 2024.
